# Single-cell transcriptome profiles the heterogeneity of tumor cells and microenvironments for different pathological endometrial cancer and identifies specific sensitive drugs

**DOI:** 10.1038/s41419-024-06960-8

**Published:** 2024-08-07

**Authors:** Fang Ren, Lingfang Wang, Yuyouye Wang, Jiaxuan Wang, Yuanpei Wang, Xiaole Song, Gong Zhang, Fangfang Nie, Shitong Lin

**Affiliations:** 1 Department of Obstetrics and Gynecology, The First Affiliated Hospital of Zhengzhou University, Zhengzhou, Henan China; 2 Zhejiang Provincial Key Laboratory of Precision Diagnosis and Therapy for Major Gynecological Diseases, Women’s Hospital, Zhejiang University School of Medicine, Hangzhou, China; 3 Department of Hepatobiliary and Pancreatic Surgery, The First Affiliated Hospital of Zhengzhou University, Zhengzhou, Henan China; 4 Department of Obstetrics and Gynecology, Union Hospital, Tongji Medical College, Huazhong University of Science and Technology, 1277 Jiefang Avenue, 430022 Wuhan, Hubei PR China

**Keywords:** Endometrial cancer, Immunogenetics

## Abstract

Endometrial cancer (EC) is a highly heterogeneous malignancy characterized by varied pathology and prognoses, and the heterogeneity of its cancer cells and the tumor microenvironment (TME) remains poorly understood. We conducted single-cell RNA sequencing (scRNA-seq) on 18 EC samples, encompassing various pathological types to delineate their specific unique transcriptional landscapes. Cancer cells from diverse pathological sources displayed distinct hallmarks labeled as immune-modulating, proliferation-modulating, and metabolism-modulating cancer cells in uterine clear cell carcinomas (UCCC), well-differentiated endometrioid endometrial carcinomas (EEC-I), and uterine serous carcinomas (USC), respectively. Cancer cells from the UCCC exhibited the greatest heterogeneity. We also identified potential effective drugs and confirmed their effectiveness using patient-derived EC organoids for each pathological group. Regarding the TME, we observed that prognostically favorable CD8^+^ Tcyto and NK cells were prominent in normal endometrium, whereas CD4^+^ Treg, CD4^+^ Tex, and CD8^+^ Tex cells dominated the tumors. CXCL3^+^ macrophages associated with M2 signature and angiogenesis were exclusively found in tumors. Prognostically relevant epithelium-specific cancer-associated fibroblasts (eCAFs) and SOD2^+^ inflammatory CAFs (iCAFs) predominated in EEC-I and UCCC groups, respectively. We also validated the oncogenic effects of SOD2^+^ iCAFs in vitro*.* Our comprehensive study has yielded deeper insights into the pathogenesis of EC, potentially facilitating personalized treatments for its varied pathological types.

## Introduction

Uterine corpus cancer ranks as the sixth most prevalent malignancy among women, with 417,000 new cases and 97,000 deaths reported in 2020. The incidence rate of endometrial cancer (EC) has increased by 132% over the past three decades, a rise that mirrors the growing prevalence of risk factors, notably obesity and an aging population [1]. Traditionally, according to Bokhman et al. (1983), EC is classified into Type I (moderately to well-differentiated EEC) and Type II based on clinical-pathological characteristics and prognosis, which includes poorly differentiated EEC (EEC-II), uterine serous carcinomas (USC), uterine clear cell carcinomas (UCCC), and uterine carcinosarcoma [2]. Our research, alongside other studies, has uncovered significant variations in epidemiological characteristics, clinical parameters and prognosis among EC samples of different pathological types [3–5]. In stark contrast to the moderate or well-differentiated EEC (85%), the 5-year survival rates for USC (45.9%), uterine carcinosarcoma (53.6%), and UCCC (63%) are notably lower, reflecting their aggressive behavior, such as cervical involvement, peritoneal and lymph-node metastasis, or distant organ metastasis [6, 7]. Currently, according to the National Comprehensive Cancer Network (NCCN) guidelines, there is no specific or effective treatment available to enhance prognosis by addressing such aggressive behavior [8]. Immunotherapy brings a glimmer of hope for treating malignant tumors, and the Food and Drug Administration (FDA) has approved pembrolizumab and Dostarlimab-gxly for advanced or recurrent EC in 2017 and 2021, respectively [9, 10]. However, the response rate of these treatments varies significantly among patients with different pathological types or tumor mutation burden (TMB) statuses. Thus, developing effective therapeutic strategies for these uncommon pathological types by thoroughly understanding their unique characteristics in malignant cells and the tumor microenvironment (TME) is crucial.

Previous studies have identified specific mutation hotspots that contribute to the development and progression of EC patients across various pathological types [11]. Notably, mutations in PTEN, PIK3CA, and PIK3R1 are associated with the progression of endometrioid EC, while mutations in TP53 and/or p53 are associated with the early development of USC [12–14]. Additionally, PTEN and TP53 mutations are common in uterine carcinosarcomas [15]. Moreover, approximately 30% of USC patients exhibit HER2 overexpression, potentially benefiting from trastuzumab therapy [16]. However, these findings are derived from bulk RNA/DNA/protein sequencing, which aggregates results from multiple cellular components, thereby obscuring the heterogeneity across and within individuals. This complexity demands further investigation using more precise techniques. With the advent of single-cell RNA sequencing (scRNA-seq), exploring the heterogeneity of tumor cells and the TME in depth has become possible. Several studies utilizing scRNA-seq technology have detailed the dynamic transitions from normal endometrium to EC and the remodeling of the TME following pembrolizumab treatment [17–19]. Some key molecules involved in the process of progression or recurrence have also been identified using the scRNA-seq technology [19, 20]. For example, Lien et al. revealed that reduced VIM in epithelial cells was significantly correlated with distant metastasis and poorer prognosis in low-stage EC samples [20]. Similarly, Cassier et al. demonstrated that Netrin-1 blockade significantly inhibits tumor growth and the epithelial-mesenchymal transition (EMT) process in EC [19]. Nonetheless, these studies predominantly focus on endometrioid adenocarcinomas, with a notable gap in research exploring the heterogeneity among EC of different pathological types via scRNA-seq, highlighting an area for future investigation.

To thoroughly investigate the unique characteristics of pathogenesis and the TME across various EC samples with different pathological types, we conducted scRNA-seq on 18 patients, including seven samples with well-differentiated endometrioid endometrial carcinomas (EEC-I), three samples with EEC-II, four samples with USC, three samples with UCCC, and one normal sample with uterine leiomyoma. Our study comprehensively elucidated the heterogeneity in percentage, functional status and cell–cell communication among different cell subpopulations, including identified malignant epithelial cells, NK_T cells, fibroblasts, macrophages and endothelial cells (ECs) using the single-cell expression atlas and in vitro experimental methods. Collectively, these findings offer valuable research resources for understanding EC pathogenesis and present novel insights for developing personalized cancer treatment for EC patients.

## Results

### The transcriptomic landscape of EC with different pathological types at the resolution of single-cell

**Fig. 1:**
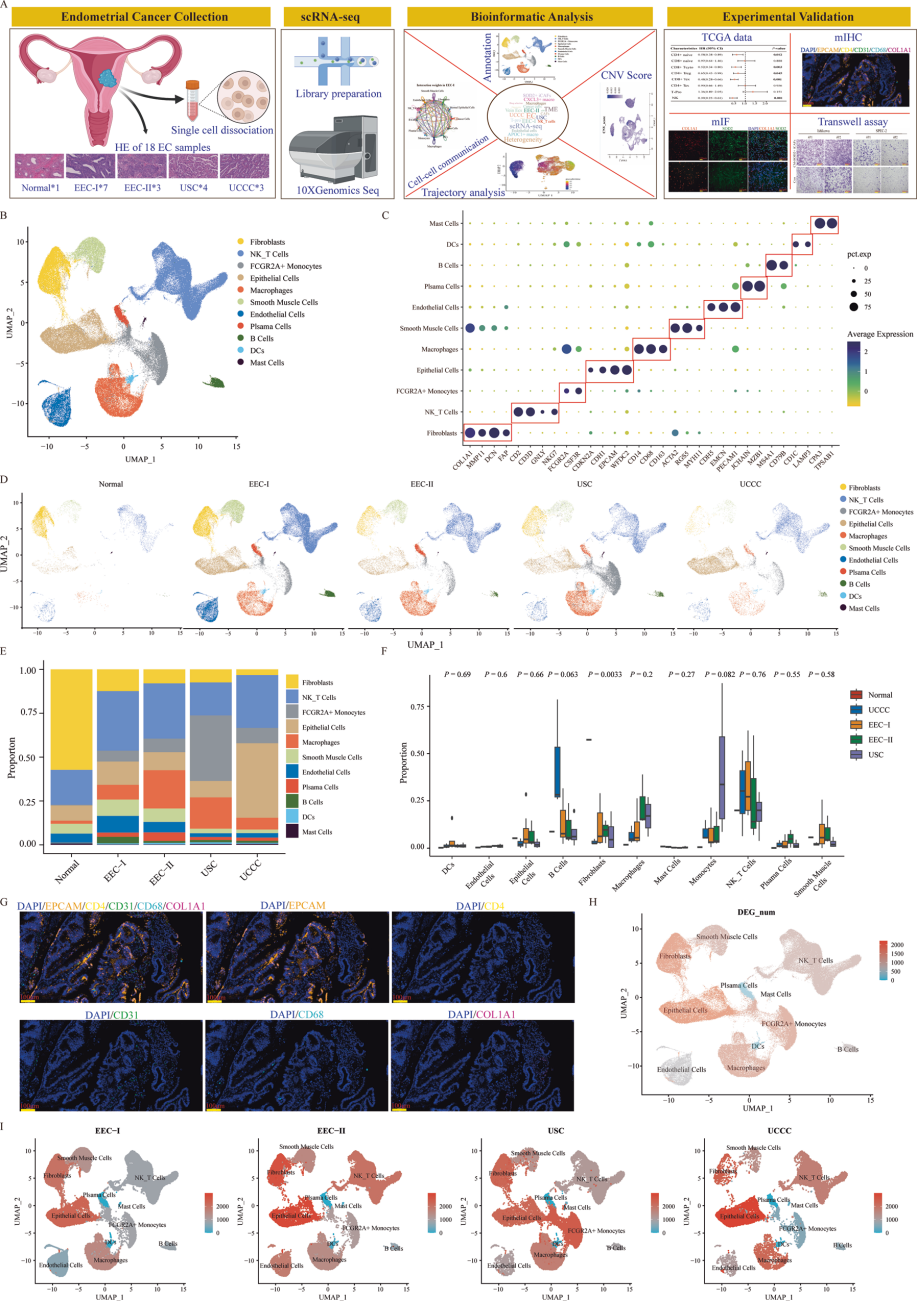
The transcriptomic landscape at the resolution of scRNA-seq from 18 EC patients with different pathological types. **A** The flowchart of this study included sample collection, scRNA-seq, bioinformatic analysis and experimental validation. **B** Uniform Manifold Approximation and Projection (UMAP) plots displayed 11 identified major cell types from 146,332 single cells. **C** Dot plots showed the normalized expression levels of marker genes in each cell cluster. **D** The distribution of each annotated cell cluster in patients with different pathological types. **E** The proportion of each annotated cell cluster in different pathological groups. **F** The comparison of the proportion of each annotated cell cluster across different pathological types. The *P*-values were calculated by the Kruskal–Wallis test. **G** Validation of certain identified cell types using the mIHC staining in one collected EC sample. **H** UMAP plots displayed the numbers of differentially expressed genes (DEGs) (|Log2FC| > 0.25, adj-*P* < 0.05) in each annotated cell cluster in all samples. The *P*-values were calculated by the Wilcoxon Rank Sum Test. **I** The numbers of DEGs (|Log2FC| > 0.25, adj-*P* < 0.05) in each annotated cell cluster from EEC-I, UCE-II, USC and UCCC groups, respectively. The *P*-values were calculated by the Wilcoxon Rank Sum Test.

To elucidate the heterogeneity of various cellular components and functional statues within the TME of EC across different pathological types, we conducted scRNA-seq on 18 samples (seven samples with EEC-I, three samples with EEC-II, four samples with USC, three samples with UCCC, and one normal sample) as shown in Fig. 1A. The clinical characteristics of the samples were presented in Supplementary Table 1. Following standard data processing procedures, including data quality control, filtering, and doublet removal, a total of 146,332 single cells were identified for subsequent analysis. We found an average of 2791 genes and 10,548 unique molecular identifiers (UMIs) per cell. Using the Seurat package for unsupervised clustering analysis and uniform manifold approximation and projection (UMAP) for visualization, we distinguished 11 major cell clusters (Fig. 1B). According to the expression of canonic marker genes, we annotated these clusters as follows: fibroblasts (17,661 cells: COL1A1, FAP, MMP11 and DCN), NK_T cells (42,362 cells: CD2, CD3D and GNLY), FCGR2A^+^ monocytes (19,659 cells: FCGR2A and CSF3R), epithelial cells (21,408 cells: CDKN2A, CDH1, EPCAM and WFDC2), macrophages (18,017 cells: CD14, CD68 and CD163), smooth muscle cells (9575 cells: ACTA2, RGS5 and MYH11), ECs (9259 cells: CDH5, EMCN and PECAM1), plasma cells (3610 cells: JCHAIN and MZB1), B cells (3245 cells: MS4A1 and CD79B), dendritic cells (DCs) (1083 cells: CD1C and LAMP3), mast cells (453 cells: CPA3 and TPSAB1) (Fig. 1C). The identified differentially expressed genes (DEGs) for each annotated cell cluster were listed in Supplementary Table 2 (|Log2FC| > 0.25, *P-*adj < 0.05, Wilcoxon Rank Sum Test). The distribution of each annotated cell cluster across different pathological groups was displayed (Fig. 1D), illustrating substantial inter-individual heterogeneity in EC samples, as further evidenced by the varying proportions of the 11 cell types across different pathological types and individual samples (Fig. 1E, F and Supplementary Fig. 1A). Furthermore, we employed multicolor immunohistochemistry (mIHC) to confirm the presence of some cell clusters by scRNA-seq in an EC sample (Fig. 1G).

We further assessed the heterogeneity within each annotated cell type by examining the gene expression profiles, specifically identifying DEGs (|Log2FC| > 0.25, *P-*adj < 0.05, Wilcoxon Rank Sum Test) across each cell cluster from varying pathological types and a normal sample. As depicted in Fig. 1H and Supplementary Fig. 1B, the epithelial cells, fibroblasts, macrophages, NK_T cells, and ECs from tumor samples were remarkably different from those from the one normal sample. Subsequent analysis showed that the epithelial cells and fibroblasts were the top two ranked cell clusters with a high number of DEGs in EEC-I/EEC-II/UCCC groups, with the exception of monocytes in the USC group (Fig. 1I and Supplementary Fig. 1C). Consequently, we concentrated on these cell types, which exhibited more DEGs, to further investigate their heterogeneity across different pathological EC types.

### Identification of malignant epithelial cells and specific chemotherapy drugs in different pathological types of EC

**Fig. 2:**
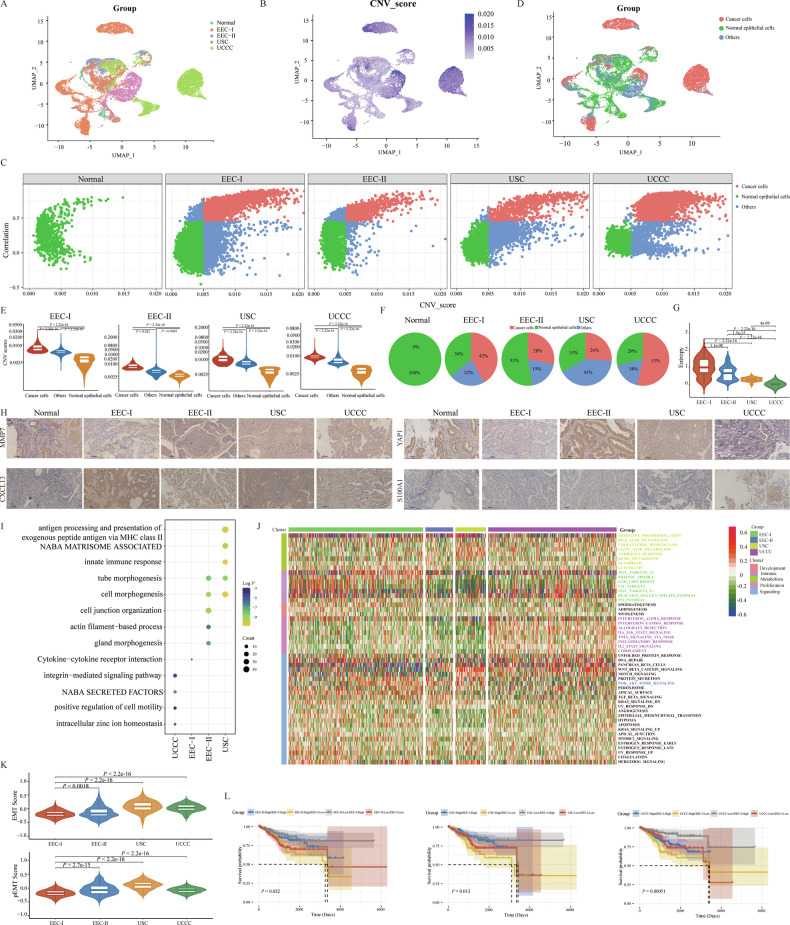
Identification of malignant epithelial cells. **A** The distribution of all epithelial cells in 18 EC samples with different pathological types. **B** UMAP plots displayed the estimated CNV score of each epithelial cell. **C** Identification of cancer cells, normal epithelial cells and others in different groups with different pathological types. **D** The distribution of cancer cells, normal epithelial cells and others in all the samples. **E** The comparison of CNV scores of cancer cells, normal epithelial cells and others in different pathological groups. The *P*-values were calculated by Student’s *t*-test. **F** The proportions of cancer cells, normal epithelial cells and others in different pathological groups, respectively. **G** Entropy analysis of cancer cells identified from different pathological groups. The *P*-values were calculated by Student’s *t*-test. **H** mIHC staining of MMP7, CXCL13, YAP1 and S100A1 in EC samples with different pathological types. **I** GO and KEGG pathway enrichment analysis of identified DEGs in different pathological groups. **J** The estimated GSVA scores of certain cancer-related pathways. **K** The estimated EMT/pEMT score in EC samples with different pathological types. The *P*-values were calculated by Student’s *t*-test. **L** Kaplan–Meier survival curves of samples with different gene signatures in the TCGA cohort. The *P*-values were calculated by the Log-rank test.

Tumor cells constitute the primary component of tumor mass and are pivotal in facilitating distant metastasis. In this study, we investigated the heterogeneity of malignant epithelial cells by analyzing their distinct transcriptional patterns across different pathological groups. Initially, we employed the “InferCNV” R Package to calculate the copy number variation (CNV) scores of 21,408 epithelial cells (Fig. 2A, B and Supplementary Fig. 1D). Following the methodology outlined by Zhang et al., epithelial cells within each pathological group were categorized into cancer cells, others, and normal epithelial cells, based on the derived CNV scores and correlation coefficients (see methods, Fig. 2C, D). The calculated CNV scores for cancer cells were notably higher compared to those for others and normal epithelial cells across the pathological groups (Fig. 2E). Additionally, the distribution of each identified epithelial cluster varied significantly among the different pathological groups (Fig. 2F). Entropy analysis showed that cancer cells from the UCCC group exhibited the lowest entropy score, indicating a substantial heterogeneity level within these cells (Fig. 2G), in contrast to the EEC-I group.

We identified DEGs in cancer cells across various pathological groups (|Log2FC| > 0.25, *P-*adj < 0.05, Wilcoxon Rank Sum Test) (Supplementary Table 3). Certain representative DEGs for identified cancer cells were displayed in Supplementary Fig. 1E: EEC-I (SGCD, KIF26B, CXCL13, and CALCB); EEC-II (IGKC, YAP1, IGLC3, and IGHG4), USC (AL357507.1, MUC4, MMP7, and AL079338.1), and UCCC (ISG15, IGFBP5, S100A1, and PI3). These genes were further validated by IHC (Fig. 2H). Functional enrichment analysis revealed that cancer cells from UCCC samples mainly got involved in the pathways of integrin-mediated signaling, NABA SECRETED FACTORS, positive regulation of cell motility, and intracellular zinc ion homeostasis. While the cancer cells from USC samples mainly got involved in the pathways of antigen processing and presentation of exogenous peptide antigen via MHC class II, NABA MATRISOME ASSOCIATED, and innate immune response. Cancer cells from EEC-I samples mainly participated in the pathway of cytokine-cytokine receptor interaction, and the cancer cells from the EEC-II samples mainly got involved in the pathways of cell junction organization, gland morphogenesis, and actin filament-based process (Fig. 2I). It is acknowledged that tumor development is linked to abnormal cells behavior acquiring cancer’s hallmarks. Gene set variation analysis (GSVA), utilizing four modules derived from the top 50 cancer-related hallmarks, showed distinct pathway activities in different pathological groups (Fig. 2J) [21, 22]. Metabolism pathways (e.g., OXIDATIVE_PHOSPHORYLATION, CHOLESTEROL_HOMEOSTASIS, FATTY ACID_METABOLISM, HEME_METABOLISM, ANDROGEN_RESPONSE) were active in USC cancer cells, termed metabolism-modulating cancer cells (Supplementary Fig. 2A). Proliferation pathways (e.g., MYC_TARGETS_V2, MITOTIC_SPINDLE, G2M_CHECKPOINT, E2F_TARGETS,) were enriched in EEC-I cancer cells, termed proliferation-modulating cancer cells (Supplementary Fig. 2B). UCCC cancer cells, which engaged in immune-related pathways (e.g., TNFA_SIGNALING_VIA_NFKB, INTERFERON_ALPHA_RESPONSE, IL6_JAK_STAT3_SIGNALING, COMPLEMENT, INFLAMMATORY_RESPONSE), were deemed immune-modulating (Supplementary Fig. 2D). The PI3K_AKT_MTOR_SIGNALING was significantly activated in EEC-II cancer cells (Supplementary Fig. 2C). Transcription factors (TFs) played crucial roles in regulating gene expression, with key TFs showing high activity and upregulated expression in cancer cells from each pathological group (Supplementary Fig. 3A, B).

Distant metastasis significantly contributes to cancer mortality globally, accounting for approximately 90% of cancer-related deaths [23]. Prior research has shown that patients from different pathological groups have varying potentials for metastasis, with those diagnosed with USC exhibiting a higher propensity for lymphatic space invasion, lymph node involvement, and microscopic diffusion on the peritoneal surface [24]. Two critical processes in metastasis, partial epithelial-to-mesenchymal transition (p-EMT) and epithelial-to-mesenchymal transition (EMT) have been identified [25, 26]. Our findings indicate that the estimated EMT and p-EMT scores in cancer cells of the USC group were significantly elevated compared to those in the other three pathological groups (Student’s *t*-test, Fig. 2K). Moreover, Kaplan–Meier survival curve analysis of the TCGA cohort showed that patients with high-EEC-I and low-EEC-II/USC/UCCC signatures had the most favorable prognosis among the subgroups analyzed (Fig. 2L).

**Fig. 3:**
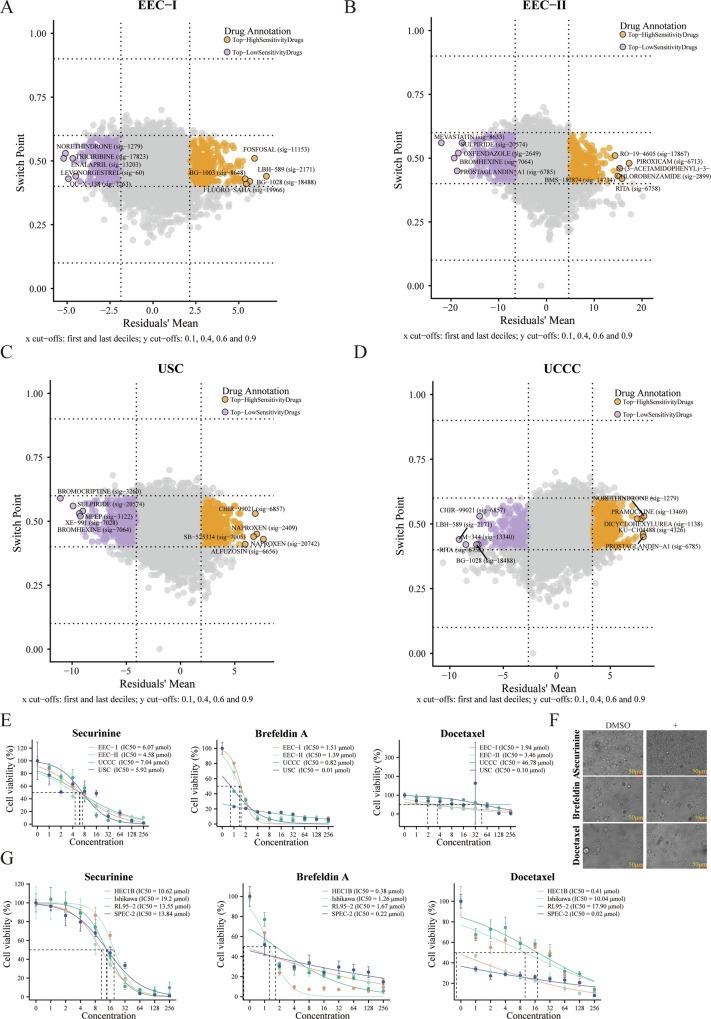
Identification of sensitive chemotherapy drugs for EC patients with different pathological types. **A** Identification of certain sensitive chemotherapy drugs for EEC-I group. **B** Identification of certain sensitive chemotherapy drugs for EEC-II group. **C** Identification of certain sensitive chemotherapy drugs for USC group. **D** Identification of certain sensitive chemotherapy drugs for UCCC group. **E** IC50 values of Securinine (left), Brefeldin (middle), and Docetaxel (right) in four EC-derived organoids. **F** The morphological changes of an EC-derived organoid after being treated with Securinine (upper), Brefeldin (middle), and Docetaxel (lower). **G** IC50 values of Securinine (left), Brefeldin (middle), and Docetaxel (right) in HEC1B, Ishikawa, RL95-2, and SPEC-2, respectively.

The findings demonstrated that cancer cells across various pathological groups exhibit diverse activities in cancer-related pathways. This diversity was attributed to their different expression profiles, potentially paving the way for new avenues in molecular pathogenesis research and the development of targeted therapeutic strategies for EC. Consequently, we identified specific chemotherapy drugs for each pathological type using the “Beyondcell” R package, based on their unique single-cell expression profiles [27]. The sensitive chemotherapy drugs identified were listed in Supplementary Table 4 and Supplementary Fig. 1F, with the top 5 drugs displaying high sensitivity across different pathological groups illustrated in Fig. 3A–D. Further validation of drug sensitivity was conducted using cell lines, and EC-derived organoids were created from collected samples in vitro. As shown in Fig. 3E, three drugs (Docetaxel, Brefeldin A, Securinine), common to each pathological group, were selected for in vitro sensitivity measurement. The morphological changes in an EC-derived organoid treated with these drugs were shown in Fig. 3F. Four different pathological EC-derived organoids displayed excellent sensitivity to Docetaxel (0.10–46.78 μmol), Brefeldin A (0.01–1.51 μmol), Securinine (4.58–7.04 μmol), which indicated their high tumor-killing ability in EC (Fig. 3E). Additionally, the half maximal inhibitory concentration (IC50) values for Docetaxel (0.02–17.90 μmol), Brefeldin A (0.22–1.67 μmol), Securinine (10.62–19.2 μmol) in four EC cell lines were also low (Supplementary Fig. 1F and Fig. 3G), underscoring the reliability of our screened drugs as evidenced by all these findings.

### Mapping the infiltration abundance and functional status of different NK_T cells in the different pathological types of EC

**Fig. 4:**
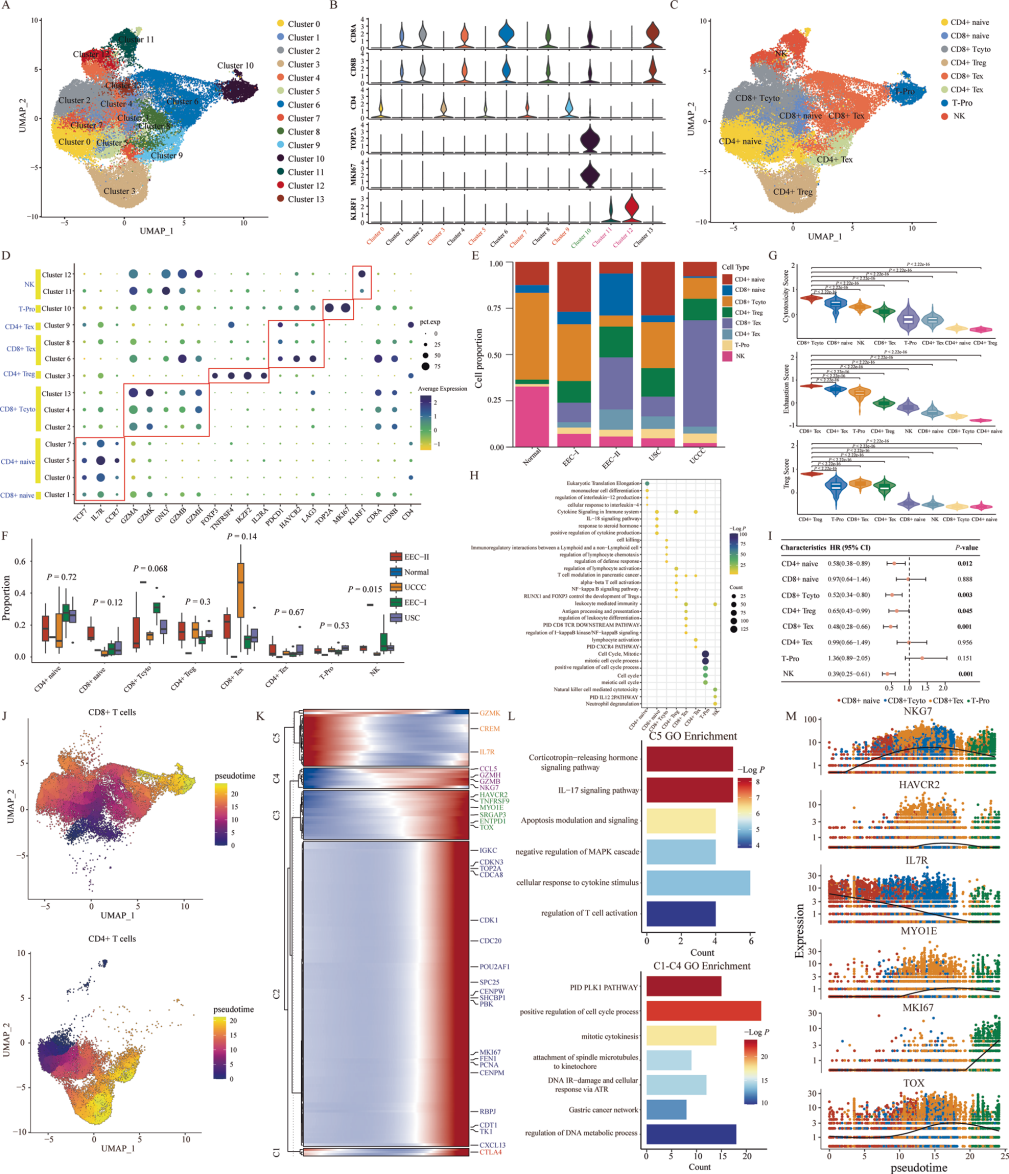
Characteristics and subpopulations of NK_T cells in EC samples with different pathological types. **A** UMAP plots displayed 14 subclusters of NK_T cells. **B** The expression levels of some marker genes in different cell clusters. **C** NK_T cells were annotated as eight major immune cell types based on different marker genes. **D** The dot plots showed the relative expression levels of marker genes in each annotated immune cell cluster. **E** The bar plot showed the cell proportion of each annotated immune cell cluster in different pathological groups. **F** The comparison of the proportion of each annotated immune cell cluster across different pathological types. The *P*-values were calculated by the Kruskal–Wallis test. **G** The estimated cytotoxicity score, exhaustion score and Treg score of each identified cell subcluster. The *P*-values were calculated by Student’s *t*-test. **H** GO and KEGG pathway enrichment analysis of DEGs for each annotated immune cell cluster. **I** The forest plot displayed the prognostic effects of each identified cell cluster on EC patients from the TCGA cohort regarding overall survival. The *P*-values were calculated by univariate Cox regression model. **J** The pseudo-time of each CD8^+^ (top) and CD4^+^ (bottom) T cell, respectively. **K** The pseudo-time heatmap displayed the dynamic changes of some involved genes in the developmental process of NK_T cells. **L** The enrichment GO terms of gene sets for different subclusters involved in the developmental process of NK_T cells. **M** The dynamic expression changes of some function-related genes (NKG7, HAVCR2, IL7R, MYO1E, MKI67, and TOX) during the pseudo-time.

NK_T cells are a critical component of the TME in EC, significantly influencing the development, progression and response to therapy of malignancies [28, 29]. We analyzed 42,362 NK_T cells, dividing them into 14 subclusters to distinguish their inherent heterogeneity among different pathological groups (Fig. 4A). Utilizing canonical marker genes, these subclusters were initially categorized into four major cell types: cluster 1/2/4/6/8/13 were CD8^+^ T cells (CD8A and CD8B), cluster 0/3/5/7/9 were CD4^+^ T cells (CD4), cluster 11/12 were NK cells (KLRF1), and cluster 10 was proliferation T cells (T-Pro) (MKI67 and TOP2A) (Fig. 4B and Supplementary Fig. 4A) [30, 31]. Further analysis based on reported marker genes categorized all CD8^+^ T cells into naïve CD8^+^ T cells (CD8^+^ naïve: TCF7, IL7R, and CCR7), cytotoxic CD8^+^ T cells (CD8^+^ Tcyto: GZMA, GZMB, GZMK, GNLY, and GZMH), exhausted CD8^+^ T cells (CD8^+^ Tex: PDCD1, HAVCR2, and LAG3). Also, CD4^+^ T cells were classified as naïve CD4^+^ T cells (CD4^+^ naive: TCF7, IL7R, and CCR7), exhausted CD4^+^ T cells (CD4^+^ Tex: PDCD1, HAVCR2, and LAG3), and regulatory CD4^+^ T cells (CD4^+^ Treg: FOXP3, TNFRSF4, IKZF2, and IL2RA) (Fig. 4C, D) [17, 18]. Cytotoxic marker genes (GZMA, GZMB, GNLY, and GZMH) were also prevalent in NK cells. Moreover, a combination of cytotoxic (GZMA. GZMB, and GZMH) and exhausted (PDCD1, HAVCR2, and LAG3) marker genes were observed in T-Pro cells, highlighting their complexity in EC specimens (Fig. 4D). The identified DEGs for NK_T subclusters were provided in Supplementary Table 5 (|Log2FC| > 0.25, *P* < 0.05, Wilcoxon Rank Sum Test). The proportions of each NK_T subcluster varied significantly across individuals and pathological groups, demonstrating extensive intra- and inter-heterogeneity in the TME (Fig. 4E, F and Supplementary Fig. 4B). Notably, NK cell proportions gradually decreased from normal to UCCC group, inverse to the trend observed in T-Pro cells. CD4^+^ Tex and CD8^+^ Tex cells were exclusive to four tumor groups, with CD8^+^ Tcyto cells being most prevalent in the normal sample.

We then explored the functional status of identified NK_T subclusters based on the well-known gene signatures [32, 33]. We found that CD8^+^ Tcyto, CD8^+^ Tex, and CD4^+^ Treg cells each obtained the highest cytotoxicity/exhaustion/Treg score, which was consistent with previous studies (Fig. 4G) [34]. We further investigated the differences of cytotoxicity score in CD8^+^ Tcyto cells, exhaustion score in CD8^+^ Tex cells, and Treg score in CD4^+^ Treg cells across different pathological groups (Supplementary Fig. 4C). Some specific TFs were also identified for each NK_T subcluster (Supplementary Fig. 4D, E). For example, KLF5 was found to be active and upregulated in naïve T cells, which has been proven to be mainly expressed in pro-T cells and is responsible for mediating TCRβ germline transcription [35]. In addition to the FOXP3, the BATF was also specifically expressed in the CD4^+^ Treg cells, which has been proven to mediate the differentiation and maturation of CD4^+^ Treg cells [36]. Functional enrichment analysis revealed that different NK_T subclusters played varied roles in functional regulation. For example, CD4^+^ naïve cells were involved in mononuclear cell differentiation, regulation of interleukin-12 production, and cellular response to interleukin-4, while the pathways of cytokine signaling in the immune system, IL-18 signaling, positive regulation of cytokine production were active in CD8^+^ naïve cells (Fig. 4H). CD8^+^ Tcyto cells mainly mediated the pathways of cell killing, regulation of defense response and regulation of lymphocyte chemotaxis, while the pathways of NF-kappa B signaling and RUNX1 and FOXP3 control the development of Tregs were active in CD4^+^ Treg. Cell cycle-related pathways (e.g., cell cycle, mitotic, positive regulation of cell cycle process) and natural killer cell-mediated cytotoxicity were remarkably active in T-Pro cells and NK cells, respectively. The pathways of PID CD8 TCR downstream and PID CXCR4 were active in CD8^+^ Tex and CD4^+^ Tex, respectively. Further prognostic analyses performed by univariate Cox regression revealed that CD4^+^ naive cells (HR = 0.58, 95% CI = 0.38–0.89, *P* = 0.012), CD8^+^ Tcyto cells (HR = 0.52, 95% CI = 0.34–0.80, *P* = 0.003), CD4^+^ Treg cells (HR = 0.65, 95% CI = 0.43–0.99, *P* = 0.045), CD8^+^ Tex cells (HR = 0.48, 95% CI = 0.28–0.66, *P* < 0.001), and NK cells (HR = 0.39, 95% CI = 0.25–0.61, *P* < 0.001) were significantly correlated with patients favorable overall survival (OS) in the TCGA cohort (Fig. 4I).

We further investigated the dynamic changes in gene expression and functions of CD4^+^ T and CD8^+^ T cells throughout their development using the Monocle3 algorithm. As shown in Fig. 4J, CD8^+^ naïve cells were identified in the early state, CD8^+^ Tcyto and CD8^+^ Tex cells in the intermediate state, and T-Pro cells in the late state. The genes that changed dynamically over pseudo-time were classified into five subclusters. Genes in cluster 1/2/3/4 exhibited gradual upregulation and mediated the pathways of PID PLK1, positive regulation of the cell cycle process, and attachment of spindle microtubules to kinetochore (Fig. 4K, L). Conversely, genes in cluster 5 were gradually downregulated during the pseudo-time, influencing pathways such as IL-17 signaling, negative regulation of MAPK cascade, and regulation of T cell activation (Fig. 4K, L). Certain representative marker genes were displayed in Fig. 4M. For example, TOX, a crucial TF in regulating CD8^+^ Tex cells, increased progressively over pseudo-time [37, 38]. Meanwhile, IL7R, vital for the differentiation, development, and maturation of T cells, decreased steadily [39]. Additionally, CD4^+^ naïve cells were found in the early stage, whereas CD4^+^ Treg and CD4^+^ Tex cells were distributed in the late stage (Fig. 4J). The genes changing dynamically over pseudo-time were also categorized into five subclusters. Genes in cluster 4/5 were downregulated, affecting pathways like positive regulation of receptor signaling pathway via JAK-STAT and immune response enhancement (Supplementary Fig. 5A, B), while those in cluster 1/2/3 were gradually upregulated over pseudo-time, involving cytokine signaling in the immune system, leukocyte activation regulation, lipopolysaccharide response. CXCL13, which was discovered predominantly at the end of Tex, exhibited a significant increase in CD4^+^ Tex cells [40]. While costimulatory (TNFRSF4) and inhibitory (CTLA4) immune checkpoints increased sharply in the CD4^+^ Treg cells (Supplementary Fig. 5C). We measured the expression of certain common immune checkpoints (CD47, CD96, CTLA4, TNFRSF1B, HAVCR2, LAG3, PDCD1, and TIGIT) across different annotated NK_T subclusters in various pathological groups. As shown in Supplementary Fig. 5D, exhausted T cells exhibited higher expression levels of these immune checkpoints, with those in the UCCC group showing the highest expression. In summary, we clearly annotated the subpopulations of NK_T cells and revealed their differences in proportion and functional status across different pathological groups, which could deepen our understanding of the TME in EC.

### Depicting the infiltration abundance and functional status of different macrophage cells in the different pathological types of EC

**Fig. 5:**
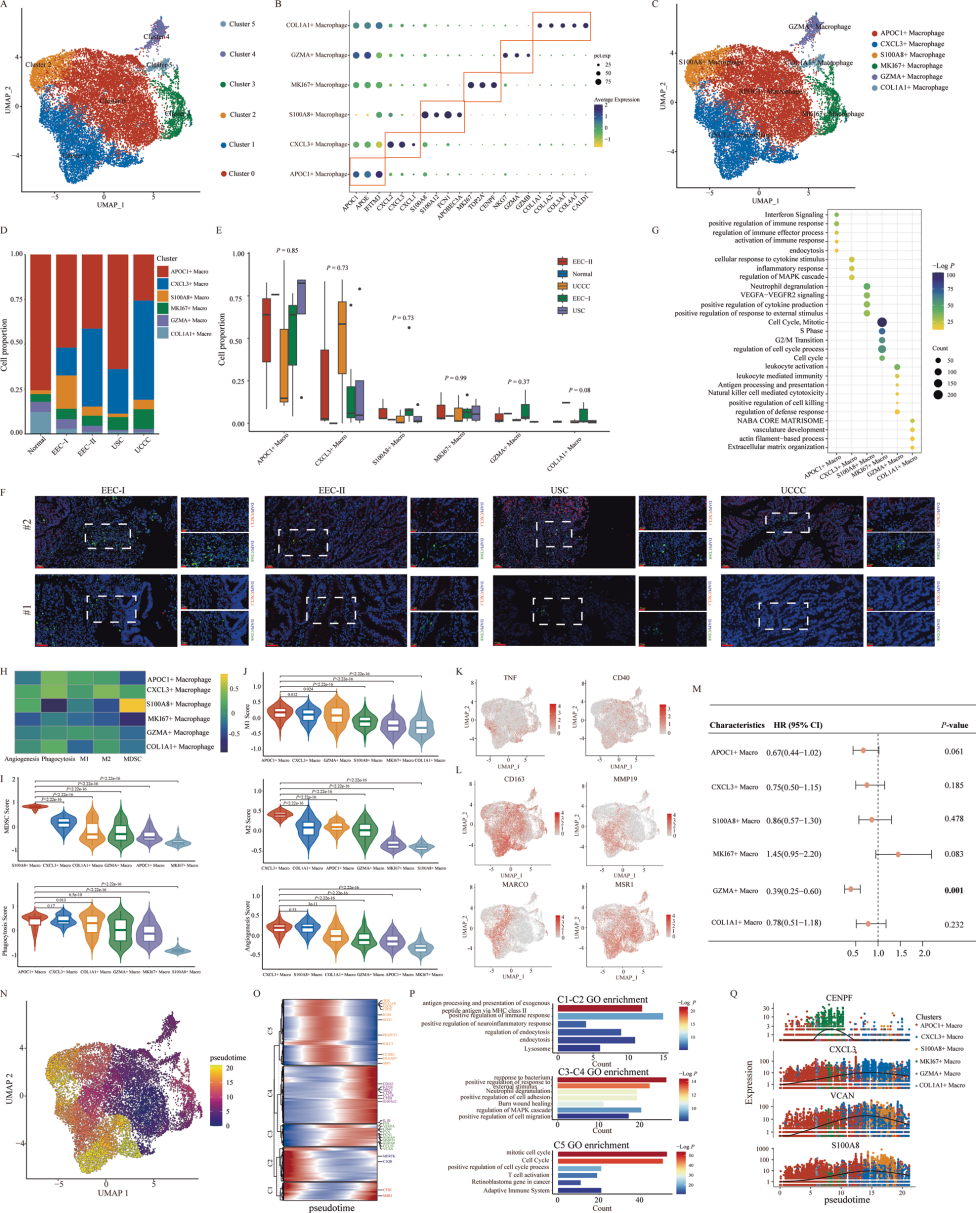
Characteristics and subpopulations of macrophage cells in EC samples with different pathological types. **A** UMAP plots displayed six subclusters of macrophage cells. **B** The expression levels of certain specific marker genes in each annotated macrophage cell cluster. **C** UMAP plots displayed the six annotated macrophage cell clusters in all samples. **D** The bar plots showed the cell proportion of each annotated macrophage cell subcluster in different pathological groups. **E** The comparison of the proportion of each annotated macrophage cell cluster across different pathological groups. The *P*-values were calculated by the Kruskal–Wallis test. **F** mIHC staining of CXCL3^+^ macrophages in different pathological groups. **G** GO and KEGG pathway enrichment analysis of DEGs in each annotated macrophage cell cluster. **H** The heatmap showed the estimated GSVA scores for different functions in each annotated macrophage cell cluster. **I**, **J** The comparison of GSVA scores for different functions across different macrophage cell clusters. The *P*-values were calculated by Student’s *t*-test. **K** The UMAP plots displayed the expression of certain marker genes (TNF and CD40) of M1-related signatures. **L** The UMAP plots displayed the expression of some marker genes (CD163, MMP19, MARCO, and MSR1) of M2-related signatures. **M** The forest plot displayed the prognostic effects of each annotated macrophage subcluster on the patients from the TCGA cohort regarding overall survival. The *P*-values were calculated by univariate Cox regression model. **N** The estimated pseudo-time of each macrophage cell. **O** Pseudo-time heatmap displayed the dynamic changes of certain involved genes in the developmental process of macrophage cells. **P** The enrichment GO terms of different gene sets involved in the developmental process of macrophage cells. **Q** The dynamic expression changes of certain function-related genes (CENPF, CXCL3, VCAN, and S100A8) during the pseudo-time.

In our study, we re-clustered a total of 18,017 macrophages cells into six subclusters (Fig. 5A). Based on marker gene expression, we annotated each cluster as a specific cell type as follows: cluster 0 as APOC1^+^ macrophage (APOC1, APOE and IFITM3), cluster 1 as CXCL3^+^ macrophage (CXCL2, CXCL3 and CXCL1), cluster 2 as S100A8^+^ macrophage (S100A8, S100A12, FCN1 and APOBEC3A), cluster 3 as MKI67^+^ macrophage (MKI67, TOP2A and CENPF), cluster 4 as GZMA^+^ macrophages (NKG7, GZMA and GZMB), and cluster 5 as COL1A1^+^ macrophage (COL1A1, COL1A2, COL3A1, COL4A1 and CALD1) (Fig. 5B, C). The estimated proportions of these macrophage subclusters varied among different samples and pathological groups (Fig. 5D, E and Supplementary Fig. 6A, B). Specifically, the proportions of COL1A1^+^ macrophages and GZMA^+^ macrophages deceased in tumor groups, while CXCL3^+^ macrophages appeared exclusively in tumor samples. We confirmed these findings using mIHC staining, which showed the presence of CXCL3^+^ macrophages and GZMA^+^ macrophages in four pathological groups (Fig. 5F and Supplementary Fig. 6D). The DEGs for each macrophage subcluster were listed in Supplementary Table 6 and Supplementary Fig. 6C (|Log2FC| > 0.25, *P* < 0.05, Wilcoxon Rank Sum Test). Functional enrichment analysis for these DEGs demonstrated that each macrophage subcluster engaged in distinct functional pathways (Fig. 5G). For instance, APOC1^+^ macrophages were primarily involved in interferon signaling and various immune response regulation pathways, including positive regulation, activation, and endocytosis. CXCL3^+^ macrophages primarily influenced cellular responses to cytokine stimulus, inflammatory response, and MAPK cascade regulation. GZMA^+^ macrophages were associated with leukocyte activation and immunity. S100A8^+^ macrophages were active in VEGFA-VEGFR2 signaling, cytokine production and external stimulus response. Pathways related to the cell cycle (cell cycle, mitotic, S phase, regulation of cell cycle process) and extracellular matrix (ECM) (NABA CORE MATRISOME, ECM organization) were predominant in MKI67^+^ and COL1A1^+^ macrophages, respectively.

Leveraging well-established gene signatures, we investigated the functional status of each annotated macrophages cluster [32, 41]. GSVA scores were calculated for different subclusters (Fig. 5H), revealing that APOC1^+^ macrophages exhibited significantly higher phagocytosis and M1 cores compared to other macrophage clusters (Fig. 5I, J). Conversely, CXCL3^+^ macrophages demonstrated the highest M2 and angiogenesis scores (Fig. 5J). Additionally, the distribution of key marker genes associated with M1 (TNF and CD40) and M2 (CD163, MMP19, MARCO, and MSR1) signatures was illustrated (Fig. 5K, L). We further assessed the prognostic impact of the identified macrophage clusters on the TCGA cohort using univariate Cox regression analysis, finding a significant correlation between GZMA^+^ Macrophages cluster and patient OS (HR = 0.39, 95% CI = 0.25–0.60, *P* < 0.001) (Fig. 5M). Specific TFs for each macrophage subcluster were also identified (Supplementary Fig. 6E, F), enhancing our understanding of their gene regulatory networks.

We utilized Monocle3 to investigate the dynamic shifts in gene expression and functionality during the transition among various macrophages subclusters. The findings indicated that the APOC1^+^ macrophage cluster was situated in the early state, whereas GZMA^+^ macrophages, MKI67^+^ macrophages and COL1A1^+^ macrophages were in the intermediate state, and CXCL3^+^ macrophages and S100A8^+^ macrophages were in the late state (Fig. 5N). Genes exhibiting dynamic changes throughout pseudo-time were also identified and further divided into five subclusters (Fig. 5O). Genes in cluster 1/2 underwent gradual downregulation, influencing pathways linked to the positive regulation of immune response, endocytosis and lysosome. In contrast, genes in cluster 3/4 displayed gradual upregulation, impacting pathways associated with the regulation of MAPK cascade, neutrophil degranulation, and the positive response to external stimulus (Fig. 5P). Genes within cluster 5 exhibited upregulation at the intermediate state during pseudo-time, influencing cell cycle, T cell activation and the adaptive immune system pathways (Fig. 5P). Certain representative changed genes were displayed in Fig. 5Q. For instance, CENPF, an important regulator of the G2 phase of the cell cycle, exhibited a marked upregulation in MKI67^+^ macrophages [42]. Meanwhile, VCAN, a key mediator of cell adhesion, proliferation, migration, and angiogenesis, showed gradual upregulation through pseudo-time [43].

### Charting the fibroblasts landscape in the TME of EC with different pathological types

**Fig. 6:**
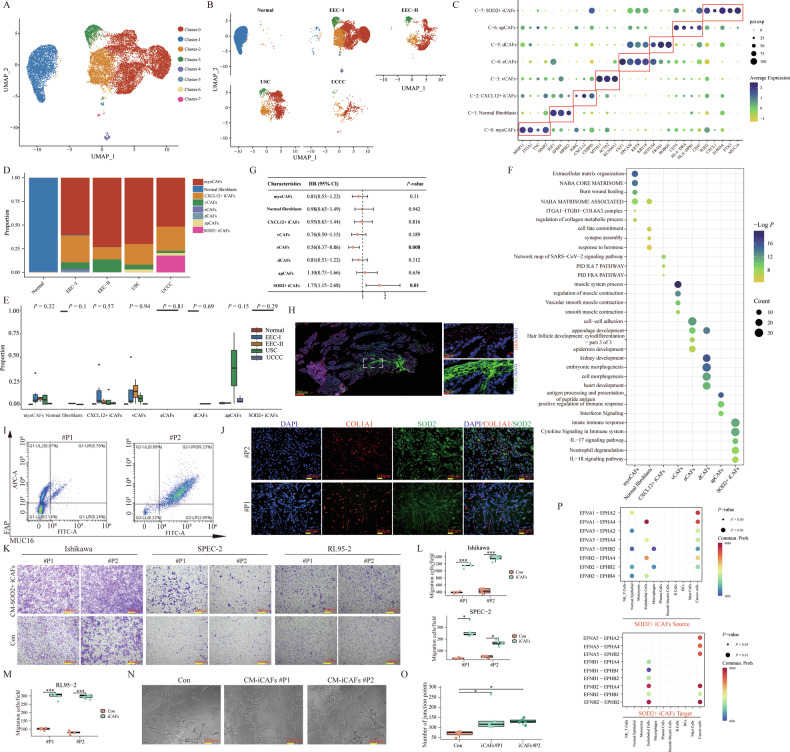
Characteristics and subpopulations of fibroblast cells in EC samples with different pathological types. **A** UMAP plots displayed the eight cell clusters of fibroblasts. **B** UMAP plots displayed the eight cell clusters of fibroblasts in different pathological groups. **C** The expression levels of certain specific marker genes in each annotated fibroblast cluster. **D** The bar plot showed the cell proportion of each annotated fibroblasts cluster in different pathological groups. **E** The comparison of the proportion of each annotated fibroblasts cluster across different pathological groups. The *P*-values were calculated by the Kruskal–Wallis test. **F** GO and KEGG pathway enrichment analysis of DEGs for each annotated fibroblasts cluster. **G** The forest plot displayed the prognostic effects of each annotated fibroblasts cluster on the patients from the TCGA cohort regarding overall survival. The *P*-values were calculated by univariate Cox regression model. **H** mIHC staining of SOD2^+^ iCAFs in one EC sample diagnosed with UCCC. **I** Extraction of SOD2^+^ iCAFs (MUC16^+^/FAP^+^) from two UCCC samples by flow cytometry. **J** Immunofluorescence staining of SOD2^+^ iCAFs sorted by flow cytometry from two UCCC samples. **K** Transwell migration assay of different EC cell lines treated with condition medium from primary SOD2^+^ iCAFs. Transwell assay had three independent biological replicates. **L**, **M** Statistical analysis of migration assay in Ishikawa, SPEC-2, and RL95-2, respectively. **P < 0.01; ***P < 0.001. The *P*-values were calculated by Student’s *t*-test. **N** Angiogenesis experiment of HUVEC treated with different condition medium from primary SOD2^+^ iCAFs or Con group. ^∗^ *P* < 0.05. Angiogenesis experiment had three independent biological replicates. **O** Statistical analysis of angiogenesis experiment in HUVEC. *P < 0.05. The *P*-values were calculated by the Kruskal–Wallis Test. **P** Bubble plots showed the communication probability of identified ligand-receptor pairs between SOD2^+^ iCAFs and other cellular components.

Cancer-associated fibroblasts (CAFs) constitute a significant element of the TME, playing a crucial role in promoting tumor progression and remodeling immunosuppression [44]. Consequently, we scrutinized the gene expression profiles and associated functions of various fibroblast subsets within EC specimens at single-cell resolution. In total, 17,661 fibroblast cells were collected, and further classified as eight subclusters (Fig. 6A). The distribution of each subcluster across different pathological groups was presented in Fig. 6B. The fibroblasts from the normal sample were distinctly segregated from those of the tumor groups, illustrating their considerable inherent heterogeneity. Based on established marker genes and tissue origins, eight subclusters were annotated as follows: cluster 0 was defined as cancer-associated myofibroblasts (myoCAFs) with high expression of MMP11, ITGA1, TNC, and MMP2 [45]; cluster 1 (IGF1, SFRP4, and HPSE2) was defined as normal fibroblasts because most of them originated from the normal sample; cluster 2 was defined as CXCL12^+^ inflammatory CAFs (iCAFs) with high expression of CXCL12, IGKC, and CEBPD [46]; cluster 3 was defined as vascular CAFs (vCAFs) with high expression of MYH11, ACTA2, and KCNMA1 [18, 47]; cluster 4 was defined as epithelium-specific CAFs (eCAFs) with high expression of CST1, EPCAM, KRT8 and KRT18 [48]; cluster 5 was defined as developmental CAFs (dCAFs) with high expression of developmentally related genes (NOTUM, FRAS1, and ROBO2) [49, 50]; cluster 6 was defined as antigen-presenting CAFs (apCAFs) with high expression of CD74, HLA-DRA, HLA-DRB1, and CD47 [48]; cluster 7 was defined as SOD2^+^ iCAFs with high expression of SOD2, CXCL1, S100A4, PTX3, and MUC16 (Fig. 6C) [46]. The relative proportions of these fibroblast subclusters varied greatly across individual samples and pathological groups, highlighting extensive intra- and inter-tumoral heterogeneity in the tumor stroma (Fig. 6D, E and Supplementary Fig. 7A). For instance, myoCAFs and CXCL12^+^ iCAFs were predominantly abundant in four tumor groups, and SOD2^+^ iCAFs were exclusively found in the UCCC group. The DEGs for each annotated fibroblast cluster were documented in Supplementary Table 7 and Supplementary Fig. 7B (|Log2FC| > 0.25, *P* < 0.05, Wilcoxon Rank Sum Test). Further GO enrichment analysis revealed that myoCAFs were primarily involved in the pathways of ECM organization, NABA CORE MATRISOME, and regulation of collagen metabolism. CXCL12^+^ iCAFs predominantly mediated the pathways of PID IL6_7 and PID FRA. Pathways involving innate immune response, IL-17/18 signaling, and neutrophil degranulation were markedly active in SOD2^+^ iCAFs. Additionally, smooth muscle (e.g., vascular smooth muscle contraction, smooth muscle contraction, muscle system process) and development (e.g., kidney development, embryonic morphogenesis, cell morphogenesis, and heart development) related signaling pathways were active in vCAFs and dCAFs, respectively. apCAFs were largely associated with pathways of antigen processing and presentation of peptide antigen, positive regulation of immune response, and interferon signaling. eCAFs primarily enhanced cell–cell adhesion, appendage development, hair follicle development, and epidermis development (Fig. 6F). Notably, within the TCGA cohort, patients exhibiting a higher SOD2^+^ iCAFs signature correlated with unfavorable OS (HR = 1.75, 95% CI = 1.15–2.68, *P* = 0.01), while eCAFs (HR = 0.56, 95% CI = 0.37–0.86, *P* = 0.008) significantly correlated with patient favorable OS (Fig. 6G). Here, we also utilized the mIHC to confirm the existence of SOD2^+^ iCAFs in one UCCC sample, and the proportion of eCAFs (EPCAM^+^/COL1A1^+^) did not seem to show significant differences between the EEC-I group and other pathological groups, which requires further validation with more samples (Fig. 6H and Supplementary Fig. 7C).

To examine the diversity within fibroblast cells, we identified certain specific TFs with high activities and expression for each subpopulation. As shown in Supplementary Fig. 7D, E, BCL6 was found specifically expressed in SOD2^+^ iCAFs, which was essential for the development of follicular helper T cell (Tfh) [51]. JDP2, more prominent in apCAFs, has been shown to impede tumor growth by modulating SDF-1 transcription in CAFs [52]. Considering the divergence of CAFs from typical fibroblasts triggered by surrounding factors, we investigated the evolutionary trajectory of CAFs in the TME [53]. As shown in Supplementary Fig. 7F, the estimated pseudo-time of each fibroblast cell was measured. The developmental trajectory was separated into two branches (Supplementary Fig. 7G), with normal fibroblasts and CXCL12^+^ CAFs positioned at the start and intermediate states of the respective branches, respectively (Supplementary Fig. 7H). In branch 1, myoCAFs and vCAFs were situated at the terminal stage. Genes with dynamic expression throughout pseudo-time in branch 1 were grouped into five clusters. Cluster 1 showed diminishing expression, while cluster 2/3 exhibited upregulation over the pseudo-time (Supplementary Fig. 7I). Functional enrichment analysis indicated that the pathways of NABA MATRISOME ASSOCIATED, NABA ECM GLYCOPROTEINS, and ECM organization were active in cluster 1. Cluster 2/3 mainly got involved in the pathways of smooth muscle contraction, burn wound healing, and blood vessel development. Meanwhile, cluster 4/5 mainly mediated the pathways of response to hypoxia and negative regulation of the immune system process (Supplementary Fig. 7J). Certain presentative dynamically changed genes were presented in Supplementary Fig. 7K. For instance, MFAP4, known to enhance vascular smooth muscle migration and proliferation, showed an increasing trend over the pseudo-time [54]. In branch 2, encompassing eCAFs, dCAFs, apCAFs and SOD2^+^ CAFs at the late stage, genes were also sectioned into five clusters (Supplementary Fig. 7H). While the expression of cluster 5 genes declined, cluster 2/3/4 saw a progressive rise throughout pseudo-time (Supplementary Fig. 7L). Functional enrichment analysis revealed that genes in cluster 2/3/4 mainly mediated the pathways of tissue morphogenesis, malignant pleural mesothelioma, and cellular response to growth factor stimulus. While genes in cluster 5 mainly mediated the pathways of NABA MATRISOME ASSOCIATED, ECM organization, and cell junction organization (Supplementary Fig. 7M). Certain presentative dynamically changed genes in branch 2 were displayed in Supplementary Fig. 7N. For instance, CST1, associated with tumor progression, and the fibroblast growth factor 4 (FGF4), imperative for embryonic development, both markedly upregulated in eCAFs and dCAFs, respectively [54, 55].

Given the adverse prognostic implications and distinctive presence of SOD2^+^ iCAFs in EC, we sought to delve into their oncogenic role in vitro. We initially extracted the primary SOD2^+^ iCAFs (MUC16^+^/FAP^+^CAFs) from two UCCC samples by flow cytometry (Fig. 6I) and further validated them using the multicolor immunofluorescence (mIF) staining (Fig. 6J). Transwell assay indicated that the conditioned medium (CM) from SOD2^+^ iCAFs could significantly promote the migrative abilities of EC cell lines (Ishikawa, SPEC-2, and RL95-2) (Fig. 6K–M). After treatment with CM from primary SOD2^+^ iCAFs, the angiogenic ability in primary human umbilical vein endothelial cell (HUVEC) cells also increased (Fig. 6N, O). Cell–cell communication analysis indicated that the L-R pairs of EFNA1-EPHA2/4, EFNA5-EPHA2/4, EFNB2-EPHA4, and EFNB2-EPHB1/2 were particularly enriched in SOD2^+^ iCAFs and other cell types, which are known to facilitate metastasis in malignant tumors (Fig. 6P) [56].

### Profiling different ECs subpopulations and their corresponding functions in different pathological types of EC

**Fig. 7:**
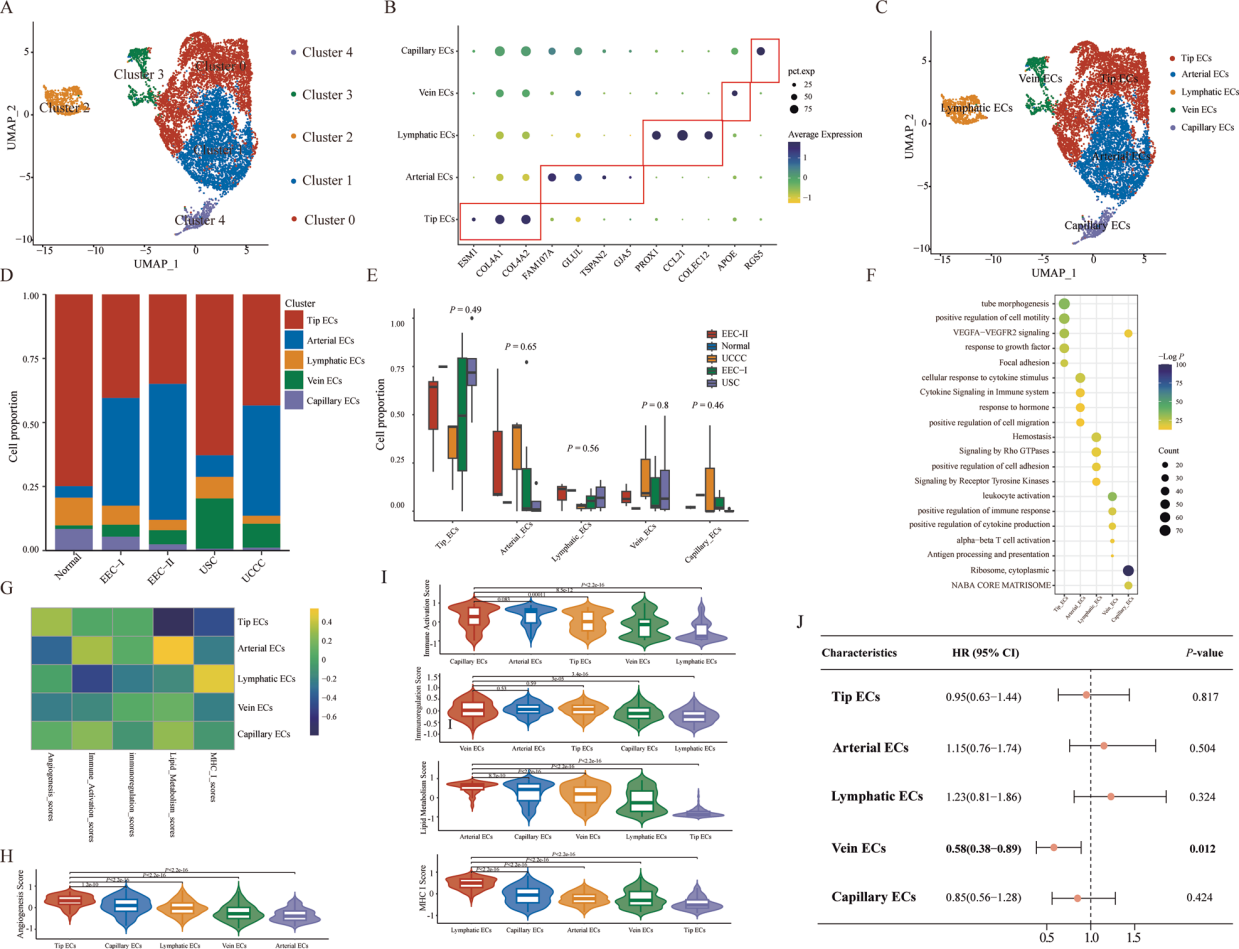
Characteristics and subpopulations of ECs in EC samples with different pathological types. **A** UMAP plots displayed five cell clusters of ECs in all samples. **B** The expression levels of certain specific marker genes in each annotated ECs cluster. **C** UMAP plots showed five annotated ECs clusters. **D** The bar plot showed the cell proportion of each annotated ECs cluster in different pathological groups. **E** The comparison of the proportion of each annotated ECs cluster across different pathological groups. The *P*-values were calculated by the Kruskal–Wallis test. **F** GO and KEGG pathway enrichment analysis of DEGs for each annotated fibroblasts cluster. **G** The heatmap showed the estimated GSVA score of different gene sets in each annotated ECs cluster. **H**, **I** The comparison of GSVA score for different functions in each annotated ECs cluster. The *P*-values were calculated by Student’s *t*-test. **J** The forest plot displayed the prognostic effects of each annotated ECs cluster on the patients from the TCGA cohort regarding overall survival. The *P*-values were calculated by univariate Cox regression model.

ECs play an important role in angiogenesis, a fundamental characteristic of tumor development and progression [57]. We divided ECs into five subclusters (Fig. 7A). Based on marker genes, we identified each subcluster as a distinct cell type: Tip ECs (ESM1, COL4A1 and COL4A2), Arterial ECs (FAM107A, GLUL, TSPAN2 and GJA5), Lymphatic ECs (PROX1, CCL21 and COLEC12), Vein ECs (APOE) and Capillary ECs (RGS5) (Fig. 7B, C) [58]. Each pathological group contained all five EC clusters, through their relative proportions varied across the different pathological groups (Fig. 7D, E). The identified DEGs of each annotated ECs cluster were detailed in Supplementary Table 8 (|Log2FC| > 0.25, *P* < 0.05, Wilcoxon Rank Sum Test). Functional enrichment analysis of DEGs within each ECs cluster indicated that different ECs subclusters served varied functions (Fig. 7F). For instance, Tip ECs were enriched in pathways involved in the positive regulation of cell motility, response to growth factors, and VEGFA-VEGFR2 signaling. Arterial ECs were implicated in pathways related to cytokine signaling in the immune system and the positive regulation of cell migration. Lymphatic ECs were engaged in pathways associated with hemostasis and signaling by Rho GTPases. The NABA CORE MATRISOME pathway was active in capillary ECs. Additionally, pathways involved in immune regulation (e.g., leukocyte activation, positive regulation of immune response, alpha-beta T cell activation) were active in vein ECs.

To investigate the functional status of each annotated ECs cluster, we calculated the GSVA scores for various known gene signatures with different functions (Fig. 7G). Results demonstrated that Tip ECs exhibited the highest angiogenesis scores (Fig. 7H). Moreover, Capillary ECs, Vein ECs, and Arterial ECs each exhibited the highest scores in immune activation, immunoregulation, and lipid metabolism respectively. Lymphatic ECs displayed significantly higher MHC-I scores compared to other ECs subclusters (Fig. 7I). Further, univariate Cox regression prognostic analyses showed a significant association between the Vein ECs cluster and favorable OS (HR = 0.58, 95% CI = 0.38–0.89, *P* = 0.012) in the TCGA cohort (Fig. 7J). This may be attributed to its heightened immunoregulatory activity, facilitating lymphocyte infiltration in the TME [59].

### Cell–cell interactions of epithelial cells with other major components in the TME

**Fig. 8:**
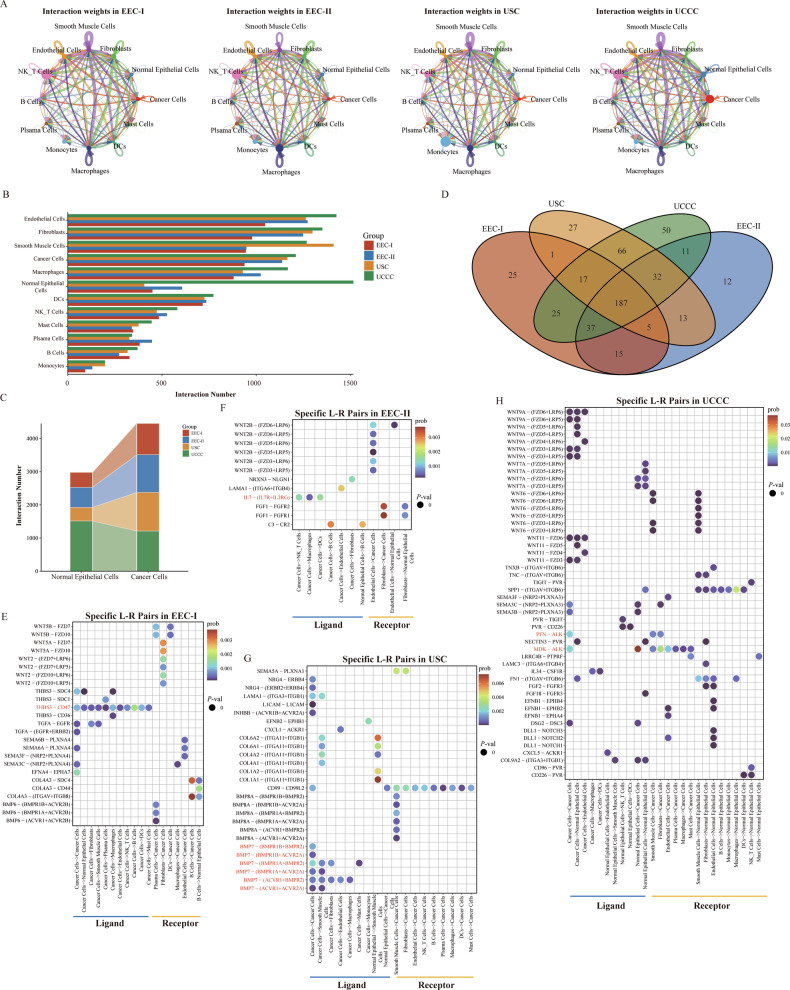
Identification of specific communication patterns between cancer cells and other cellular components in different pathological groups. **A** Interaction weights in EEC-I, EEC-II, USC, and UCCC groups, respectively. The color of lines represented the cell types. **B** Bar plot displayed the number of significant cell–cell interactions among each annotated cell type of 18 EC samples. **C** Interaction numbers of normal epithelial cells and cancer cells in EEC-I, EEC-II, USC, and UCCC groups. **D** Venn plot displayed the numbers of specific cell–cell interactions in EEC-I, EEC-II, USC, and UCCC groups, respectively. **E** Bubble plots displayed the specific interaction in EEC-I group. **F** Bubble plots displayed the specific interaction in EEC-II group. **G** Bubble plots displayed the specific interaction in USC group. **H** Bubble plots displayed the specific interaction in UCCC group.

To examine the diversity of cell–cell communications among various cellular components across different pathological groups, the CellChat database was employed to predict potential ligand-receptor (L-R) pairs. As shown in Fig. 8A, B, the total number of L-R pairs in each annotated cell type and the interaction weights between different cell types in each pathological group varied. As the epithelial cells (cancer cells and normal epithelial cells) were the major components of tumor mass and played pivotal roles in the regulation of distant metastasis and drug treatments, we mainly focused on the specific L-R pairs between cancer cells and other cellular components for subsequent analysis. As shown in Fig. 8C, cancer cells from EEC-I, EEC-II, USC groups obtained more L-R pairs than normal epithelial cells, while normal epithelial cells obtained more L-R pairs than cancer cells in UCCC group. We further identified the specific L-R pairs for each pathological group, and 25, 12, 27 and 50 L-R pairs were identified for EEC-I, EEC-II, USC and UCCC groups, respectively (Fig. 8D). As shown in Fig. 8E, we found the L-R pair of THBS3-CD47 specifically existed in EEC-I group, which is highly expressed in cancer stem cells and promotes EMT transition in malignant tumors [60, 61]. The L-R pair of IL7-(IL7R + IL2RG) was enriched between cancer cells and immune cells in EEC-II group, which plays a crucial role in the survival and differentiation of lymphocytes via IL7 receptor (Fig. 8F) [62]. The L-R pairs of BMP family proteins were remarkably enriched in UCCC group, which belong to the TGFβ family of cytokines (Fig. 8G) [63]. The L-R pairs of PTN/MDK-ALK shared the highest communication probability in USC group, which has been proven to be closely related to the occurrence of various tumors (Fig. 8H) [64]. At present, many targeted drugs have been developed for the above-specific L-R pairs, such as lemzoparlimab for CD47, and aletinib for ALK. The heterogeneity in cell–cell communication combined with specific expression profiles could provide insights into developing more specific and effective treatments for different pathological patients.

## Discussion

In this study, we presented an unprecedented transcription map of EC by conducting scRNA-seq on 18 EC samples, including some rare pathological types (poorly differentiated EEC, USC, and UCCC). This marks our study as the first to include EC samples with these rare pathological types using the scRNA-seq technology, offering an excellent resource for exploring the extensive heterogeneity of carcinogenesis and the TME across different pathological types. We systematically described the heterogeneity in proportion and functional status of cancer cells and other cellular components (macrophages, NK_T cells, fibroblasts, ECs) in the TME among different pathological groups. Furthermore, we employed various experimental methods in vitro (e.g., mIF, transwell migration assay, angiogenesis experiment, construction of EC-derived organoids, etc.) to validate our findings.

EC has increasingly become one of the most prevalent gynecological malignancies, attributed to lifestyle modification and improvements in living standards in developed countries [65]. Most patients with EC obtain favorable prognoses due to early diagnosis and timely treatment. Nonetheless, a minority of EC patients, particularly those with rare pathological types such as UCCC, USC, or uterine mixed carcinoma, tend to develop recurrence or distant metastasis due to the inherent characteristics of these types. Our research has shown that patients with these rare pathological types are older, present with a more advanced clinical stage, and have a higher incidence of myometrial infiltration, cervical involvement, lymph node metastasis, and positive ascites cytology in comparison to those with EEC-I [4, 5]. Consequently, there is a clinical necessity for an in-depth investigation into the heterogeneity of carcinogenesis. Intra-tumoral heterogeneity presents a significant global research challenge, mainly because of its genetic variations that propel the advancement of human cancer and contribute to drug resistance development, thus being a critical factor in the failure of drug treatments [66]. In our study, we performed the Entropy analysis to assess the homogeneity of cancer cells within pathological groups. We found that cancer cells from the UCCC or USC groups showed significantly lower Entropy scores than those from the EEC-I group, suggesting greater heterogeneity among cancer cells. High heterogeneity may impair the immune system’s ability to combat cancer and facilitate tumor cell immune escape, eventually leading to tumor progression (recurrence or distant metastasis) [67]. Therefore, patients with UCCC or USC have a significantly worse prognosis than those with EEC-I [4, 5].

Cancer cells from different pathological groups also displayed different activities in cancer hallmarks. We found that the pathways related to proliferation were significantly enriched in the cancer cells of the EEC-I group. In previous studies, Liu et al. also conducted GSEA analysis on EC samples and identified five significant pathways that may affect EC progression, including E2F targets, G2M checkpoint, mTORC1 signaling, MYC targets v1 and MYC targets v2. Among them, the G2M checkpoint was a part of the cell cycle pathway and has been proven to be most relevant to EC prognosis. Moreover, certain related genes on the cell cycle pathway were also associated with EC prognosis [68]. The PI3K_AKT_MTOR_SIGNALING pathway was significantly activated in the cancer cells from the EEC-II group in our study. Studies have shown that in type II EC, phosphorylation-dependent oncogenic signaling in the phosphatidylinositol-4,5-bisphosphate 3-kinase (PI3K) pathway was found to be most frequently altered in type II ECs [69]. Increased PI3K/AKT/mTOR signaling was associated with disease progression and poor prognosis in patients, and may play a role in targeted therapy [70]. The GLYCOLYSIS pathway related to metabolism was found to be active in the cancer cells of the USC group. Glucose metabolism may be associated with acquired resistance paclitaxel in USC, suggesting that changes in metabolic pathways may influence the progression and treatment of USC [71]. Previous studies have also shown genes involved in immune response have been found to be highly expressed in UCCC samples, which was consistent with our results that the immune-related pathways were active in the cancer cells from the UCCC group [72]. In our study, we further predicted the sensitivity of chemotherapy drugs based on cancer cells expression profiles for each pathological group. We validated the sensitivities of potential drugs using EC cell lines and EC-derived organoids. Together, these findings may indicate that different pathological EC types have different regulatory mechanisms in disease initiation and progression, and our findings may contribute to the development of targeted treatments for specific tumor cells in each pathological group.

The cellular composition of stromal and immune cells within the TME plays a critical role in the onset and progression of EC. Elucidating their interplay may enhance the effectiveness of tumor immunotherapy [17]. Immunotherapy has provided promising treatment strategies for various solid tumors, including EC, with significant advancements following the approval of pembrolizumab for advanced or recurrent EC [73, 74]. Yet, the proportion of EC patients deriving benefit from immunotherapy is far from satisfactory, underscoring the need for a more profound understanding of TME regulation across different EC pathologies. In our study, the abundance of fibroblasts was reduced in tumor samples compared to the normal tissue, with the UCCC group exhibiting the lowest proportion. We also observed effector cells (e.g., NK cells and CD8^+^ Tcyto) significantly correlated with EC favorable prognosis were plentiful in the normal sample, whereases the level of exhausted (CD8^+^ Tex and CD4^+^ Tex) and Treg cells (CD4^+^ Treg) increased in tumor samples. This suggests that immune exhaustion and escape are inevitable during EC carcinogenesis. Moreover, the specific proportions and functional statues of NK_T cell subpopulations significantly varied across different pathological EC types, indicating distinct regulatory mechanisms in the TME. For example, the higher proportions of exhausted cells in the ECC-II and UCCC groups indicated the acquiring state of T cell exhaustion under chronic tumor stimulation within the TME. In addition, the estimated exhaustion score of CD8^+^ Tex cells and Treg score of CD4^+^ Treg cells were significantly lower in EEC-I group than in the other pathological groups, which may indicate a more active anti-tumor activity. In fact, CD8^+^ Tex cells at different periods (e.g., pre-treatment, early on-treatment, late on-treatment) have different indications for immunotherapy [75]. Also, certain key immune checkpoints (including HAVCR2, CTLA-4, and PDCD1) also exhibited uneven distribution among the pathological groups. These differences in the TME characteristics imply that immunotherapy may differ among various EC pathologies, supporting the development of more personalized treatment plans.

CAFs, as pivotal cellular components within the TME, exhibit significant heterogeneity in terms of origin, phenotype and function across various subtypes [76]. Beyond the normal fibroblasts, our study distinctly annotated seven CAFs subtypes. Some of these subtypes (e.g., SOD2^+^ iCAFs, eCAFs, dCAFs) were not identified in prior single-cell databases of EC samples, potentially due to the inclusion of broader array of pathological types in our sequencing cohort [17, 18, 77]. Notably, certain CAFs subtypes demonstrated significant tissue specificity, for example, eCAFs and dCAFs were exclusively identified in the EEC-I group, whereas SOD2^+^ iCAFs were uniquely present in the UCCC group, which was further confirmed by mIHC in clinical samples. Additionally, eCAFs and SOD2^+^ CAFs exhibited contrasting prognostic impacts on OS in the EC cohort from the TCGA database. Through a series of in vitro experiments, we validated the cancer-promoting effects of SOD2^+^ iCAFs on EC, aligning with the functions of previously described iCAFs [78]. The intricate functional mechanisms and cell–cell communication networks between specific CAFs subtypes and other EC components warrant further investigation in vitro and in vivo. Moreover, our insights into CAFs subtypes could pave the way for the comprehensive development of CAFs targeted therapies.

Our study comprehensively maps the transcriptome landscape and provides detailed annotations, offering a valuable resource for further investigation into the carcinogenesis of EC, particularly in rare pathological types. However, several limitations were identified that require future address. First, additional EC samples of rare pathological types need to be included for scRNA-seq and subsequent validation. Second, the regulatory networks among different cellular components warrant further exploration and validation. Third, it is imperative to delve deeper into the primary causes of intra-tumoral heterogeneity at the transcriptional level across various pathological groups, such as driver mutations or epigenetic alterations. Lastly, the potential benefits of these findings for EC patients through the development of target-specific drugs need confirmation, supported by more extensive model validation both in vitro and in vivo.

In summary, this study thoroughly characterized transcriptome heterogeneity in tumor cells and the TME across various pathological types of EC samples. This has enriched our understanding of EC pathogenesis and progression, potentially aiding in the development of personalized treatments for diverse pathological types.

## Methods

### Ethical approvement and EC samples collection

This study was approved by the Medical Ethics Committee of First Affiliated Hospital of Zhengzhou University (Approved number: 2022-KY-1173-002). All the tumor tissues were collected from those patients diagnosed with different pathological types of EC who underwent comprehensive surgical staging of EC in the Department of Obstetrics and Gynecology of First Affiliated Hospital of Zhengzhou University. One normal tissue was collected from a patient diagnosed with uterine adenomyosis. All patients were treatment-naïve prior to undergoing surgical treatment. The detailed clinical characteristics of the included patients are provided in Supplementary Table 1.

### Preparation of single-cell suspensions from EC and normal tissues

The fresh ex vivo tissues were stored in sCelLiveTM Tissue Preservation Solution (Singleron, China) at 4 °C. After the specimen was transported to the laboratory, it was first washed with pre-cooling 1x phosphate-buffered saline (PBS) buffer (Servicebio, China), then cut into 2–4 mm pieces using the sterile equipment, and digested with a mixed digestive solution containing collagenase I/II (ThermoFisherScientific, USA) and DNase (Sigma, USA) for 30 min at 37 °C. Subsequently, we used a 70 μm cell filter (Biosharp, China) to obtain the single-cell suspensions, which were further lysed with the red blood cell lysis buffer (Servicebio, China) and washed with pre-cooling 1x PBS buffer (Servicebio, China) twice.

### Construction of gene expression library and scRNA-seq

The detailed methods were provided by 10x Genomics, and the used scRNA-seq data was provided by BerryGenomics (Beijing, China). Briefly, single-cell suspensions were mixed with gel beads containing barcodes, enzymes and separated oil beads containing label information to form gel beads-in-emulsion (GEMs) using the Chromium Single Cell Library, Gel Bead & Multiplex Kit (10× Genomics). In each GEM, the mRNA released by the ruptured cells was reverse-transcribed into cDNA with a barcode. Then, the oil droplets burst, and the cDNA was further collected for purification, amplification, fragment screening, and quality inspection. Finally, the cDNA was interrupted and sequenced, allowing for the construction of a second-generation sequencing library. Once the constructed library passed the quality inspection, PE150 sequencing was performed using the Illumina NovaSeq 6000 platform, with 150 bp on each end. Among them, Read1 contained Barcode and UMI double label sequences, while Read2 was the transcriptional sequence.

### scRNA-seq data preprocessing

The raw reads were aligned to the genome (Human, GRCh38.p12) using Cell Ranger (v7.0.0) for gene quantification. Subsequent data quality control, dimensionality reduction, clustering, and annotation analysis were performed using the “Seurat” R package (v4.0) [79].

### Quality control, batch effect correction, and doublet remove of scRNA-seq data

First, cells with low quality need to be removed to ensure the reliability of subsequent analyses, which contained the following characteristics: (1) less than 200 or more than 7500 genes; (2) >10% of mitochondrial genes; (3) more than 100,000 reads. Then, doublet removal was carried out using the “DoubletFinder” R package [80]. Finally, all cells from included samples were integrated using the IntegrateData function in Seurat” R package, which was used to correct the batch effect. In summary, a dataset containing 146,332 cells and 33,415 genes was obtained after the above processing. To ensure that all features contribute equally to subsequent analysis, the dataset was further scaled, and the SCTransform function was applied to perform data standardization.

### Unsupervised clustering and dimensionality reduction

Principal Component Analysis (PCA), a dimensionality reduction technique, was performed using the 2000 highly variable genes selected by FindVariableFeatures function. Thirty principal components (PCs) were calculated and used for UMAP, a powerful technique for non-linear dimensionality reduction and visualization. To further refine the understanding of cell relationships, the FindNeighbors function was employed to establish a weighted graph of cellular proximity in the reduced-dimensional space. Subsequently, the FindClusters function was applied to perform unsupervised clustering analysis based on the calculated neighbors. The clusters were determined with a specified resolution parameter of 0.5, which adjusts the granularity of cluster assignments.

### Cell type annotation based on marker genes

The process of cell type annotation was conducted with a meticulous approach, leveraging not only the identification of 23 distinct cell clusters, including 146,332 cells and 33,415 genes but also incorporating additional details for a comprehensive understanding. The annotation was achieved through an examination of marker gene expression patterns, as well as distinctive features observed in the UMAP visualization. The detailed marker genes used in our study were as follows: fibroblasts (COL1A1, MMP11, FAP and DCN), NK_T cells (CD2, CD3D and GNLY), FCGR2A^+^ monocytes (FCGR2A and CSF3R), epithelial cells (CDKN2A, CDH1, EPCAM and WFDC2), macrophages (CD14, CD68 and CD163), smooth muscle cells (ACTA2, RGS5 and MYH11), ECs (CDH5, EMCN and PECAM1), plasma cells (JCHAIN and MZB1), B cells (MS4A1 and CD79B), DCs (CD1C and LAMP3), mast cells (CPA3 and TPSAB1). The spatial distribution of cells within the UMAP plots offered additional insights into the relationships and similarities among different clusters.

### Calculation of CNV and identification of malignant epithelial cells

The methods of identifying malignant epithelial cells from all epithelial cells have been well described in the previous study [81]. Initially, a reference set comprising 2000 randomly sampled NK_T cells was used, and all epithelial cells were collected to determine their CNV scores. The process of measuring CNV was conducted using the “InferCNV” R package (v1.6.0 https://github.com/broadinstitute/inferCNV) with a refined strategy. Second, the average expression characteristics of the top 5% cells in each pathological group based on the CNV scores were also separately calculated. The correlation coefficients between each other epithelial cell and the top 5% epithelial cells within each subgroup were then calculated. Finally, all epithelial cells were categorized as “cancer cells”, “normal epithelial cells”, or “others” based on the CNV scores and correlation coefficients. The cutoff values were set as follows: CNV score greater than 0.005, and correlation coefficient greater than 0.45. In summary, this method rigorously compared the expression intensity of genes at various chromosomal positions. This detailed evaluation significantly enhanced the accuracy and precision of CNV analysis, thus facilitating a comprehensive identification and description of CNV features within the epithelial cells.

### Identification of DEGs and functional enrichment of identified cell clusters

The DEGs of each identified cell cluster were generated by FindAllMarker and FindMarkers function in Seurat” R package. The genes were identified as DEGs according to the following cutoff: adjusted *P*-value < 0.05 and |Log2FC| > 0.25. Functional enrichment analysis of identified DEGs was performed using the Metascape (https://metascape.org) [82]. Some functional gene sets of NK_T cells, macrophages and ECs were displayed in Supplementary Table 9 [32, 41].

### Survival analysis

Transcriptome expression profiles and clinical data of EC samples were downloaded from the TCGA database (https://www.cancer.gov/tcga) using the “TCGAbiolinks” R packages. The estimated score for each patient based on the identified DEGs was calculated by the GSVA, and patients were further stratified into high and low groups based on the median GSVA score. The Kaplan–Meier method was used to generate survival curves, and the Log-rank test was used to test whether the difference was significant. The univariate Cox proportional hazards regression model was used to calculate the HR. The endpoint was OS. Survival curves were drawn using the “survival” (https://github.com/therneau/survival) and “survminer” R packages (https://github.com/kassambara/survminer). The detailed clinical characteristics of included EC from the TCGA cohort were provided in Supplementary Table 10.

### Pseudo-time trajectory

The pseudo-time trajectory analysis was performed using the Monocle3 algorithm [83]. The Seurat object was translated for compatibility with “Monocle3” R package using the seurat_to_monocle3 function. The pseudo-time trajectory was learned using the learn_graph function, while cell ordering was performed with order_cells function. Monocle-specific genes were identified using the graph_test function. A subset of genes exhibiting dynamic changes during the process were selected, with q-value less than 0.05 and Moran’s I value exceeding 0.3. The pseudo-time heatmaps displayed the dynamic changes of genes were drawn by “ClusterGVis” R package.

### Identification of sensitive drugs

The potential sensitive drugs were identified by the “Beyondcell” R package for Perturbation Sensitivity (PSc) analysis [27]. In brief, the Perturbation Sensitivity scores were calculated by the bcScore function based on the gene expression matrix. After the PSc analysis, UMAP was applied to visualize cellular relationships, which was performed by bcUMAP function. The spatial distribution of cells was understood according to their gene expression profiles. To further characterize the dataset and pinpoint potential therapeutic targets, condition-based statistics and un-extended therapeutic cluster-based statistics were obtained. Specifically, the bcRanks function was used to conduct inter-group differential analysis with default parameters. Subsequently, the bc4Squares function showcased the analysis outcomes, emphasizing the top five drugs with the highest average variance. Additionally, a heatmap was employed to illustrate the Beyondcell scores of the top 20 resistant genes’ residuals within each group across various cells.

### Analysis of TFs activity and expression

PySCENIC was used to perform the single-cell regulatory network inference and clustering (SCENIC), which identified the specific assessment of TFs activity by the co-expression patterns of genes [84]. Essentially, within pySCENIC, TFs activity was evaluated by analyzing gene co-expression patterns within individual cells, particularly focusing on genes associated with TFs. This process identified genes co-expressed with specific TFs within individual cells, allowing inference on the activity status of these TFs. This analytical approach utilized correlations and expression patterns of genes to infer potential activity and regulatory networks of TFs within individual cells. PySCENIC used the count matrix of expression levels as input to calculate the co-expression modules and evaluated the weight between TFs and their target genes, in which GRNBoost algorithm was chosen. Then, TFs with direct targets (regulons) were identified using the RcisTarget and the activity of each regulon in each cell was evaluated using AUCell. Outputs of pySCENIC were input to calculate the average regulon activity (AUC) scores using “AUCell” and “SCENIC” R packages.

### Construction of cell–cell communication networks

In order to investigate the intricate communication network among various cell types, a comprehensive cell–cell communication analysis was performed using the “CellChat” R package [85]. The estimation of communication probabilities was achieved by integrating the gene expression matrix with existing knowledge regarding interactions among signaling ligands, receptors, and their cofactors. The interactions of ligand-receptor pairs with *P*-value less than 0.05 were retained. This integrated approach provided a holistic understanding of the intricate communication landscape between different cell types within the biological system.

### Culture of cell lines and preparation of CM

EC cell lines Ishikawa, RL95-2, and SPEC-2 were obtained from the American Type Culture Collection (ATCC). Primary HUVEC cell was purchased from the company of iCell Bioscience Inc (#h110). All cells were cultured with different medium (Ishikawa and SPEC-2: DMEM; RL95-2: DMEM/F-12; HUVEC: ECM) containing 10% FBS and 1% penicillin-streptomycin at the cell incubator with 37 °C and 5% CO_2_.

The detailed methods of collected CM have been well described in our previous study [33]. In brief, primary SOD2^+^ iCAFs obtained from cell sorting were cultured with complete DMEM for 48 h. Then the supernatant was collected and centrifuged at 1000×*g* for 5 min and filtered with 0.45 μm filters. All collected CM was stored at −80 °C for future use.

### IC50

First, 2000 EC cells were seeded into each well of the 96-well plate for 24 h. Then, various drugs with different solubility levels to be tested were added for 48 h, and the cell viability was measured using the Cell Counting Kit-8 (CCK-8, Dojindo Laboratories, #CK04). The IC50 value of each drug was calculated using the GraphPad Prism (version 6). The details of used drugs were as follows: Docetaxel (MCE, H-B0011), Brefeldin A (MCE, HY-16592), Securinine (MCE, HY-N2079).

We employed the same methods used for cervical cancer to construct EC-derived organoids in this study, which had been well described in our previous manuscript [33]. Once the first-generation organoids were successfully constructed, they were further collected and digested into single cells. For this, 2000 single cells were seeded into the 96-well plate and cultured for 72 h. Then, different concentrations of testing drugs (Docetaxel, Brefeldin A, and Securinine) were added for 120 h. Finally, the metabolic APT levels were calculated using the CellTiter-Glo 3D Viability Assay (Promega, #G9683) according to the manufacturer’s instructions. The morphological changes of organoids were measured using an inverted fluorescence microscope after being treated with different drugs.

### Flow-sorting for specific primary fibroblasts

The primary fibroblasts were extracted from two patients diagnosed with EC, and the method has been well described in our previous study [33]. We further performed cell sorting to collect SOD2^+^ iCAFs. The details of the used flow cytometry antibodies were as follows: anti-FAP (Abcam, ab317555), and anti-MUC16 (Abcam, ab134093).

### Transwell migration assay

For this, 40,000 EC cells (Ishikawa, RL95-2, and SPEC-2) suspended with 200 μl serum-free medium were added to the upper chamber, and 500 μl CM or complete medium was added to the lower chamber. After co-cultivating for 24 h, the lower chamber was subjected to paraformaldehyde fixation for 10 min and crystal violet staining for 30 min. The number of migrated cells was then measured using ImageJ software.

### mIF

mIF staining was performed to measure the purity of SOD2^+^ iCAFs sorted by the flow cytometry. First, the cell slides were fixed with 4% paraformaldehyde for 30 min, and the membrane was broken with 0.1% Triton100 and incubated at room temperature for 20 min. Then the cell slides were incubated with 3% BSA at room temperature for 30 min, and the primary antibodies were added and incubated at 4 °C overnight. On the next day, the secondary antibody was added and cell slides were incubated at room temperature for 1 h. Finally, DAPI staining solution was added at room temperature in the dark for 10 min. The details of used antibodies were as follows: COL1A1 (CST, #72026, 1:100), and SOD2 (Proteintech, #CL594-66474, 1:100) (Supplementary Table 11).

### Angiogenesis experiment

For this, 50 μl Matrigel (Corning, #356243) was added to each well of the 96-well plate and stored in the cell incubator at 37 °C for 30 min. After that, 20,000 HUVEC cells suspended with 100 μl complete DMEM or CM were added. The vascular microtubule was recorded at different time points and further measured using the ImageJ software.

### Hematoxylin and eosin staining (HE), and mIHC

We utilized HE staining for histopathological evaluation. Briefly, the paraffin-embedded sections went through a series of procedures, including dewaxing, dehydration, and hematoxylin and eosin staining. The detailed methodologies of mIHC and mIF have been thoroughly explained in our previous manuscript [33]. All the images were obtained by the 3D panoramic scanner (DANJIER, HISHTECH Pannoramic 250, Jinan, China) and further visualized using the CaseViewer software. The detailed information of primary antibodies used in our study was as follows: EPCAM (Abcam, ab223582, 1:500), CD68 (Abcam, ab955, 1:1000), CD4 (Abcam, ab133616, 1:600), COL1A1(CST, #72026, 1:100), CD31 (Abcam, ab28364, 1:50), CXCL1 (Proteintech, #12335-1-AP, 1:100), GZMA (Proteintech, #11288-1-AP, 1:100) (Supplementary Table 11).

### Statistical analysis

Statistical analyses were performed using the R statistical software version 4.1.0 including Wilcoxon’s rank sum test, Wilcoxon signed-rank test, and Student’s *t*-test. All statistical tests were two-sided. *P* < 0.05 was considered statistically significant.

### Supplementary information

Supplementary Figures

Supplementary Tables

## Data Availability

The scRNA-seq data of included samples has been uploaded to the Genome Sequence Archive (GSA) of the National Genomics Data Center (Access Link: https://ngdc.cncb.ac.cn/gsa-human/browse/; ID: HRA006322).
