# Supplementary material for: Single-cell transcriptome profiles the heterogeneity of tumor cells and microenvironments for different pathological endometrial cancer and identifies specific sensitive drugs
Source: Cell Death Dis. 2024 Aug 7;15(8):571. doi: 10.1038/s41419-024-06960-8 (PMC11306564; doi:10.1038/s41419-024-06960-8)
Supplement: Supplementary file 1 — Supplementary Figures [file 41419_2024_6960_MOESM1_ESM.pdf]

## Supplementary Figures

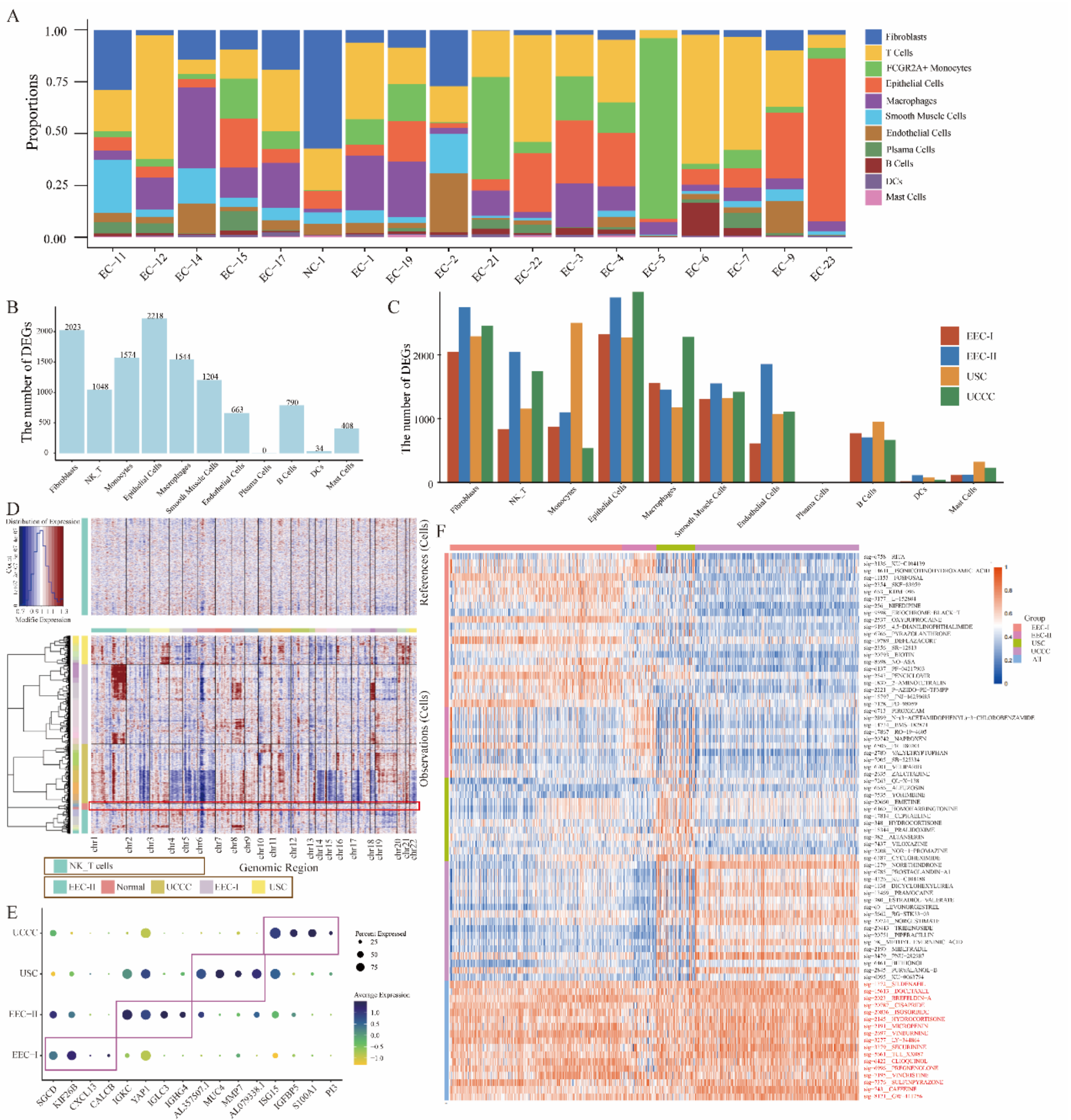


**Supplementary Figure 1. Characteristics of identified cell subpopulations. (A)** The proportions of different annotated cell clusters in each sample. **(B)** The bar plot displayed the total number of DEGs (|Log2FC| > 0.25, adj-*P* < 0.05) in each annotated cell cluster between EC samples and the normal reference. The *P*-values were calculated by Wilcoxon Rank Sum Test. **(C)** The bar plot displayed the number of DEGs (|Log2FC| > 0.25, adj-*P* < 0.05) in each annotated cell cluster between different pathological samples and the normal reference. The *P*-values were calculated by Wilcoxon Rank Sum Test. **(D)** Inferring of copy number variation (CNV) of all epithelial cells. The upper panel displayed the CNVs of reference cells (NK_T cells). The lower panel exhibited the observed CNVs of each annotated epithelial cell in

different pathological groups. **(E)** The dot plots showed the average expression levels of certain marker genes in the cancer cells from different pathological groups. **(F)** The heatmap displays specific and common chemotherapy drugs that have high sensitivity for different pathological groups.

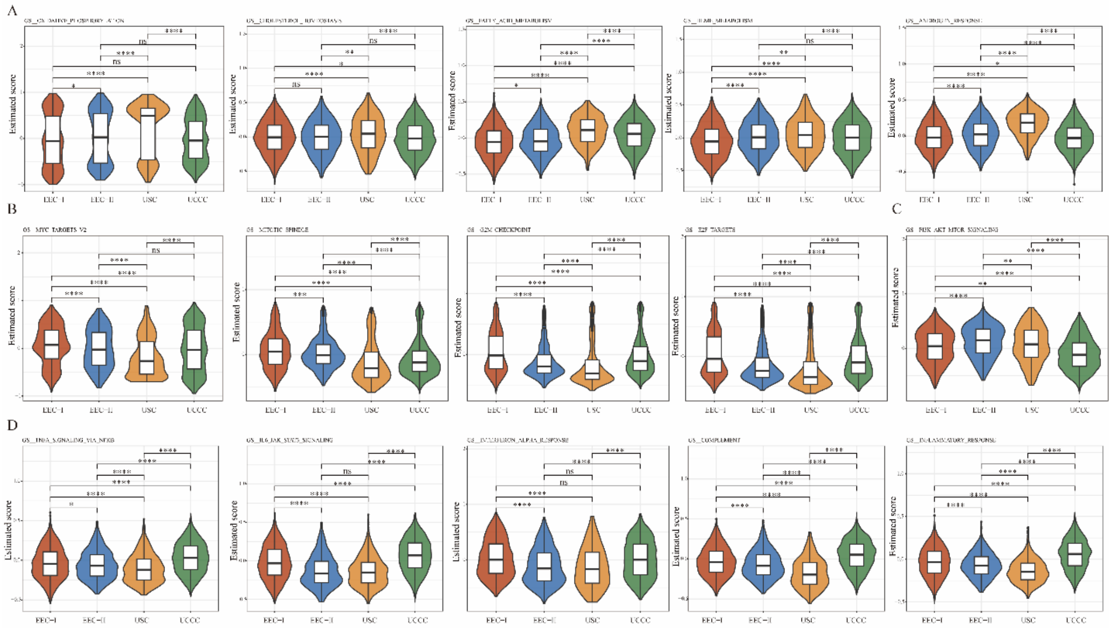


**Supplementary Figure 2. Estimated scores of some cancer hallmarks for cancer cells from different pathological groups. (A)** Estimated scores of metabolism-related pathways for cancer cells from different pathological groups. **(B)** Estimated scores of proliferation-related pathways for cancer cells from different pathological groups. **(C)** Estimated scores of PI3K_AKT-MTOR_SIGNALING for cancer cells from different pathological groups. **(D)** Estimated scores of immune-related pathways for cancer cells from different pathological groups. * $P$ < 0.05; **$P$ < 0.01; ****$P$ < 0.0001; ns not significant. The $P$-values were calculated by Wilcoxon Rank Sum Test.

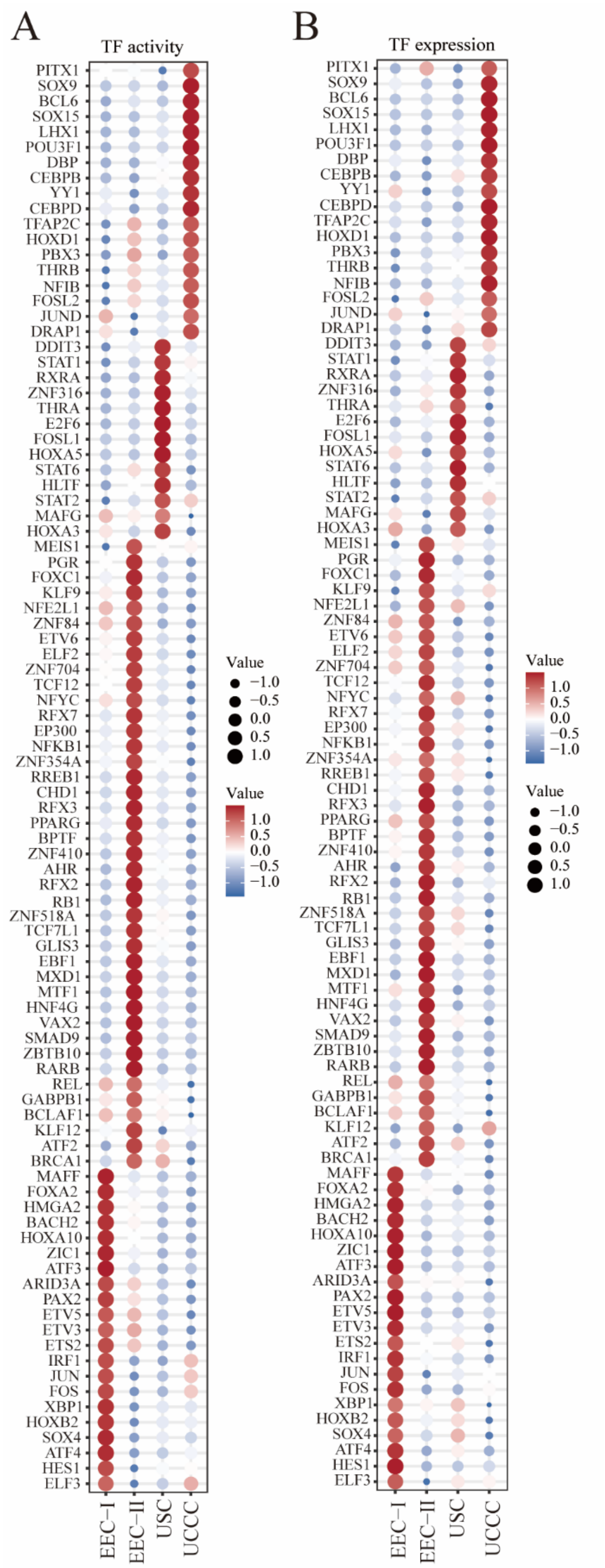


**Supplementary Figure 3. Identification of specific transcription factors (TFs) for cancer cells in each pathological group. (A)** The relative transcriptional activity of certain identified TFs across different pathological groups. **(B)** The relative expression levels of certain identified TFs across different pathological groups.

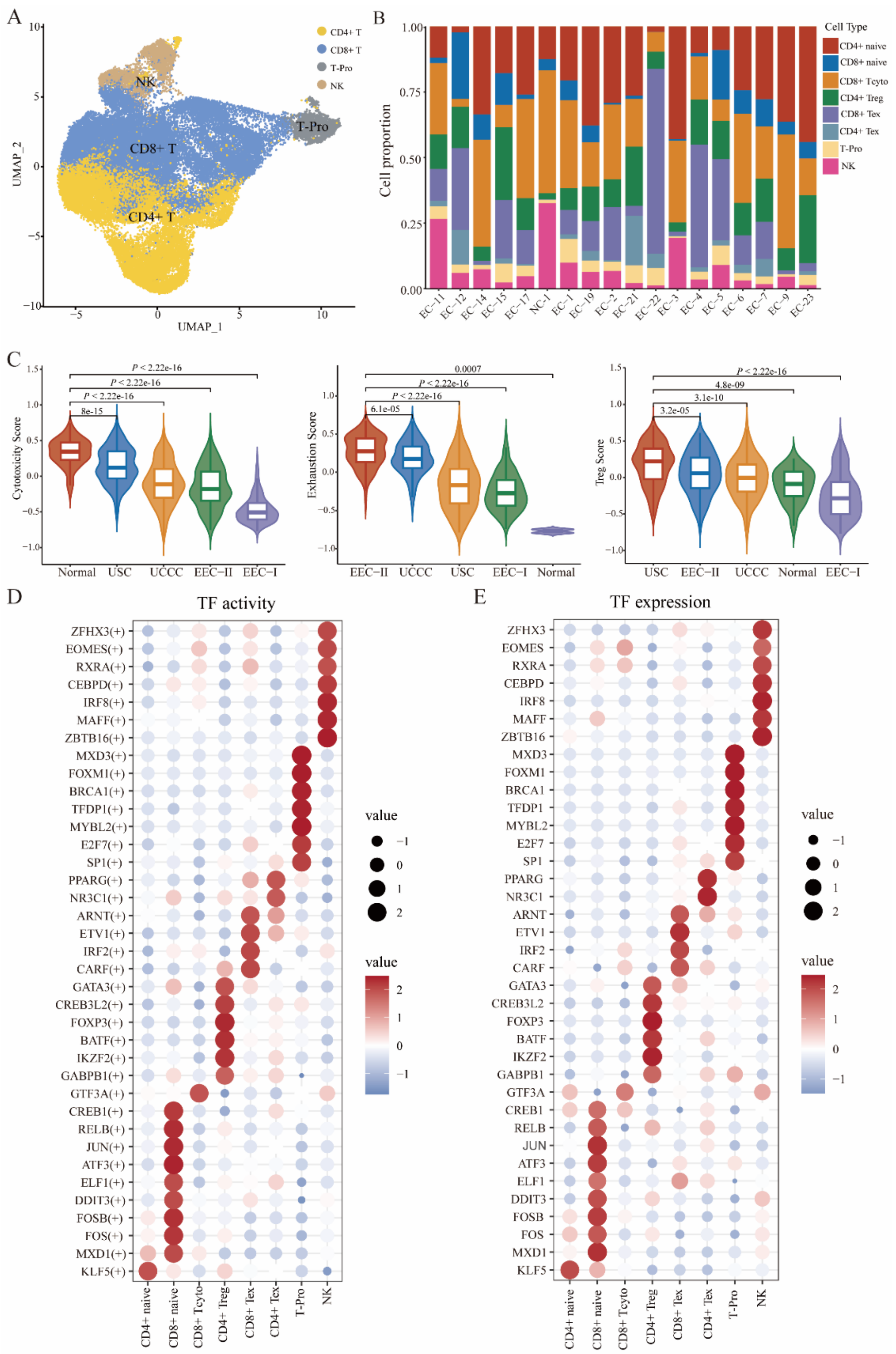


**Supplementary Figure 4. TFs analysis for different NK_T cell subpopulations in EC samples. (A)** UMAP plots displayed four major annotated cell types: CD4$^+$ T cells, CD8$^+$ T cells, proliferating T cells and NK cells. **(B)** The bar chart displayed the proportion of eight annotated immune cell clusters in each included sample. **(C)** The estimated cytotoxicity score, exhaustion score and Treg score of CD8$^+$ Tcyto cells (right), CD8$^+$ Tex cells (middle), and CD4$^+$ Treg cells (left), respectively. The *P*-values

were calculated by Student's t-test. **(D)** The relative transcriptional activity of certain identified TFs in different annotated immune cell clusters. **(E)** The relative transcriptional expression levels of certain identified TFs in different annotated immune cell clusters.

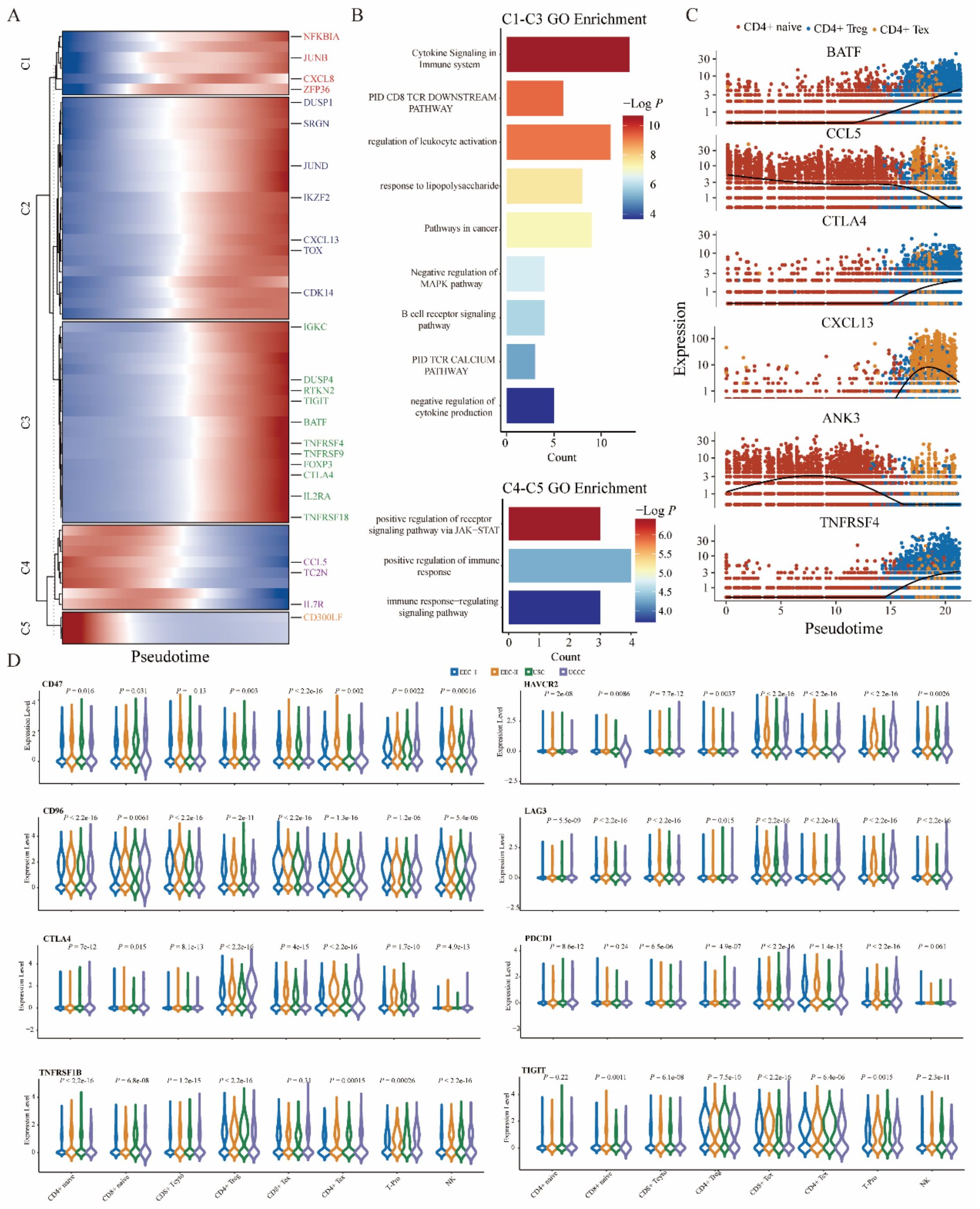


**Supplementary Figure 5. Pseudo-time trajectory analysis of CD4$^{+}$ T subpopulations. (A)** The pseudo-time heatmap displayed the dynamic changes of certain genes involved in the developmental process of CD4$^{+}$ T cells. **(B)** The

enrichment GO terms of certain gene sets involved in the developmental process of CD4$^{+}$ T cells. **(C)** The dynamic expression changes of certain function-related genes (BATF, CCL5, CTLA4, CXCL13, ANK3 and TNFRSF4) during the pseudo-time. **(D)** The relative expression levels of certain immune checkpoints in each annotated NK_T cell subpopulation across different pathological groups. The *P*-values were calculated by ANOVA.

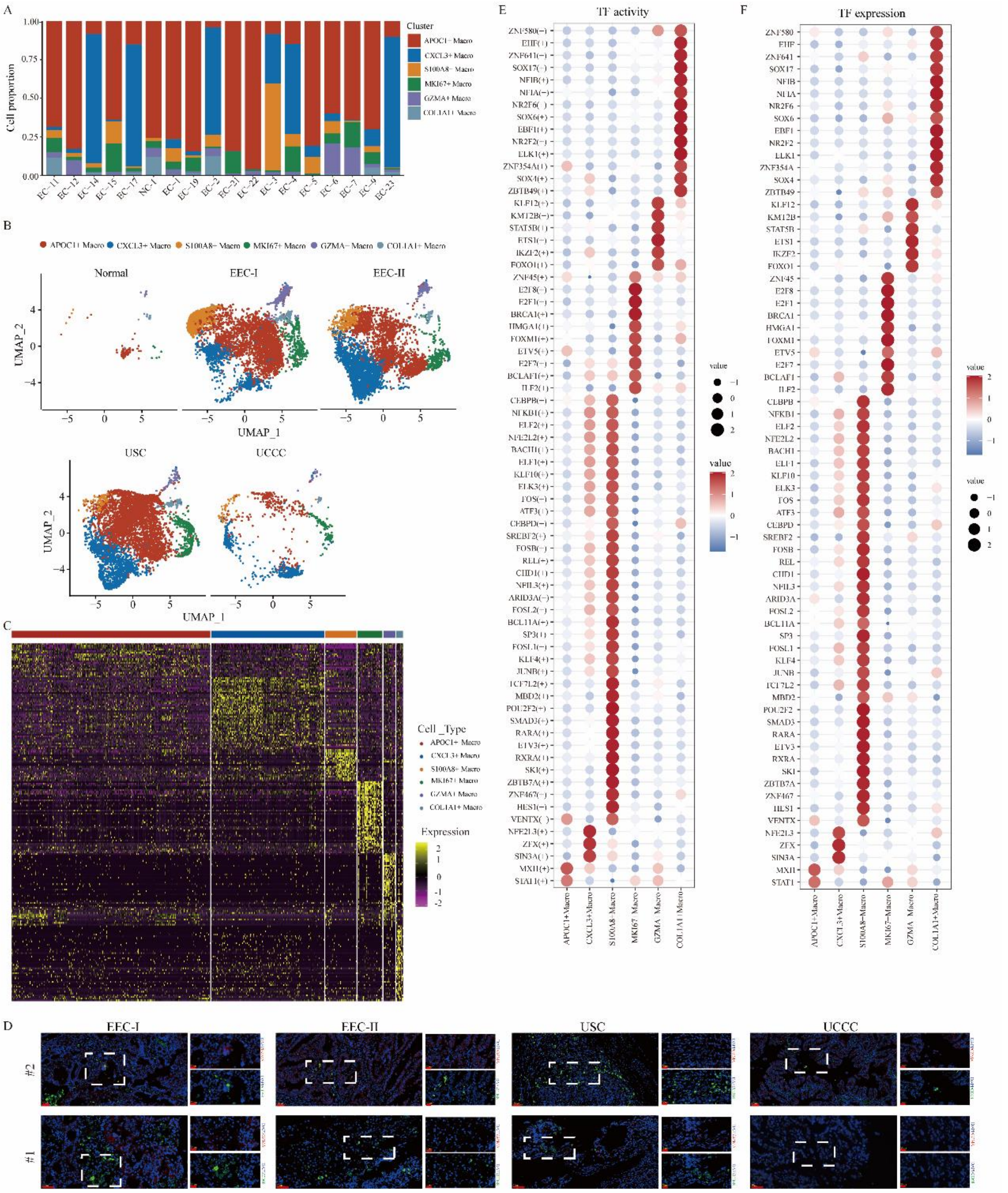


**Supplementary Figure 6. Characteristics of macrophages in EC samples. (A)** The bar chart displayed the proportions of six annotated macrophages cell clusters in each

sample. **(B)** UMAP plots displayed the distribution of six macrophages cell clusters in different pathological groups. **(C)** The heatmap displayed the expression levels of identified DEGs (|Log2FC| > 0.25, adj-$P$ < 0.05) for each macrophage subpopulation. The $P$-values were calculated by Wilcoxon Rank Sum Test. **(D)** mIHC staining of GZMA$^{+}$ macrophages in different pathological groups. **(E)** The relative transcriptional activity of certain identified TFs in different annotated macrophages cell clusters. **(F)** The relative expression levels of certain identified TFs in different annotated macrophages cell clusters.

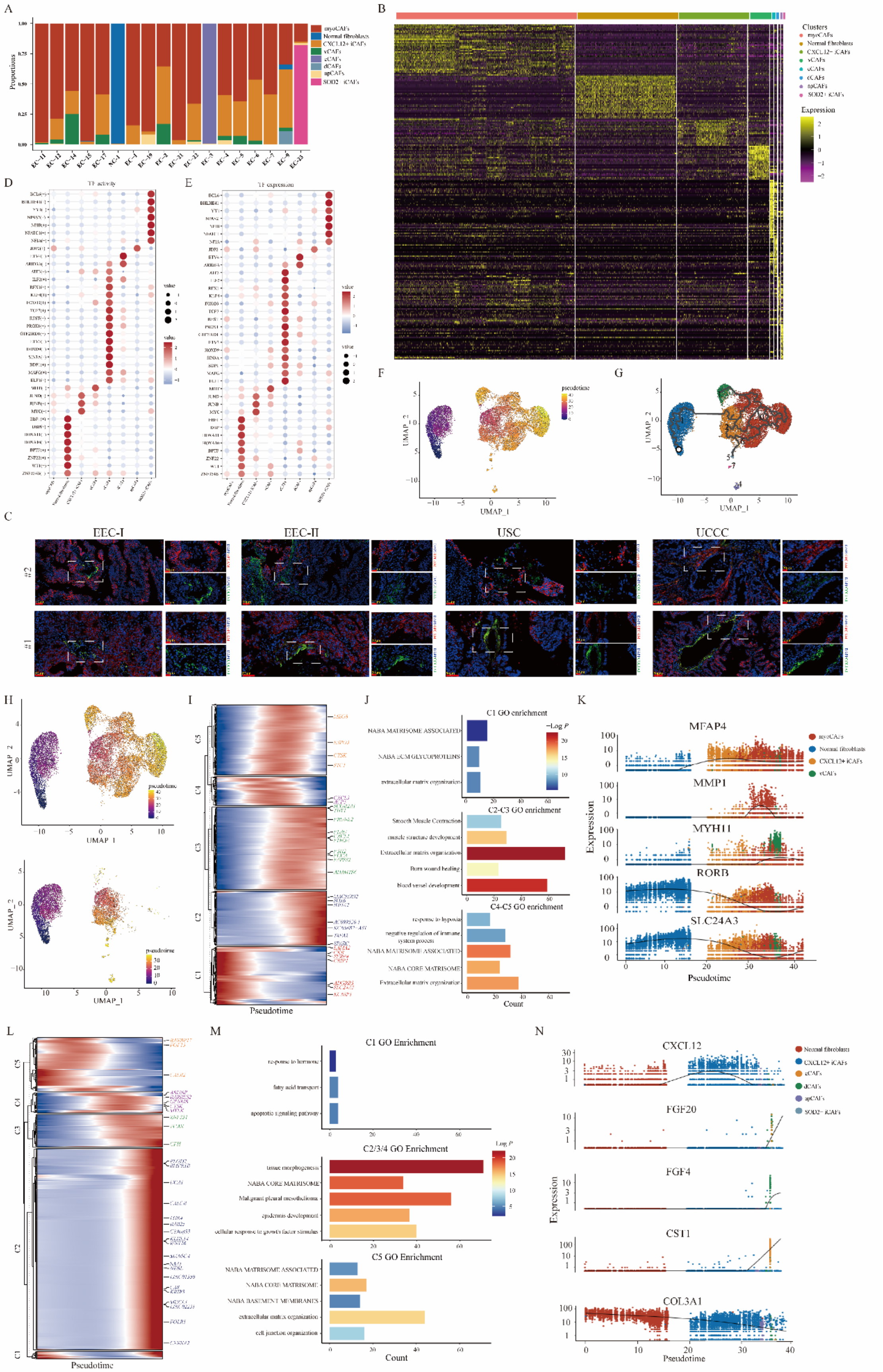


**Supplementary Figure 7. Characteristics of different identified fibroblast subpopulations in EC samples. (A)** The proportions of different annotated fibroblast

clusters in each sample. **(B)** The heatmap displayed the expression levels of certain identified DEGs (|Log2FC| > 0.25, adj-*P* < 0.05) in each fibroblast subcluster. The *P*-values were calculated by Wilcoxon Rank Sum Test. **(C)** mIHC staining of eCAFs ($EPCAM^+$/$COL1A1^+$) in different pathological groups. **(D)** The relative transcriptional activity of certain identified TFs in different annotated fibroblasts clusters. **(E)** The relative expression levels of certain identified TFs in different annotated fibroblasts clusters. **(F)** The estimated pseudo-time of each fibroblast cell. **(G)** The trajectory of the all identified fibroblast cells. **(H)** The pseudo-time of fibroblast cells identified in trajectory branch 1 (top) and trajectory branch 2 (bottom). **(I)** The pseudo-time heatmap displayed the dynamic changes of certain genes involved in the developmental process of fibroblasts in trajectory branch 1. **(J)** The enrichment GO terms of different gene sets involved in the developmental process of fibroblast cells in trajectory branch 1. **(K)** The dynamic expression changes of some function-related genes (MFAP4, MMP1, MYH11, RORB, and SLC24A3) during the pseudo-time in trajectory branch 1. **(L)** The pseudo-time heatmap displayed the dynamic changes of certain genes involved in the developmental process of fibroblasts in trajectory branch 2. **(M)** The enrichment GO terms of different gene sets involved in the developmental process of fibroblast cells in trajectory branch 2. **(N)** The dynamic expression changes of certain function-related genes (CXCL12, FGF20, FGF4, CST1, and COL3A1) during the pseudo-time in trajectory branch 2.
